# Study of the colonic epithelial-mesenchymal dialogue through establishment of two activated or not mesenchymal cell lines: Activated and resting ones differentially modulate colonocytes in co-culture

**DOI:** 10.1371/journal.pone.0273858

**Published:** 2022-08-30

**Authors:** Pascale Plaisancié, Charline Buisson, Edwin Fouché, Pierre Martin, Céline Noirot, Claire Maslo, Jacques Dupuy, Françoise Guéraud, Fabrice Pierre

**Affiliations:** 1 Toxalim (Research Centre in Food Toxicology), Toulouse University, INRAE UMR 1331, ENVT, INP-Purpan, UPS, Toulouse, France; 2 Plateforme bio-informatique GenoToul, MIAT, INRAE, UR875 Mathématiques et Informatique Appliquées Toulouse, Castanet-Tolosan, France; University of Illinois at Chicago, UNITED STATES

## Abstract

Continuous and rapid renewal of the colonic epithelium is crucial to resist the plethora of luminal deleterious agents. Subepithelial fibroblasts contribute to this turnover by regulating epithelial proliferation and differentiation. However, when intestinal homeostasis is disturbed, fibroblasts can acquire an activated phenotype and play a major role in the progression of intestinal pathologies. To evaluate the involvement of fibroblasts in the regulation of colonocytes under homeostatic or pathological conditions, we established resting and activated conditionally immortalized fibroblast cell lines (nF and mF) from mouse colonic mucosa. We then studied the epithelial-mesenchymal interactions between activated or resting fibroblasts and the normal mouse colonocytes (Co) using a co-culture model. Both fibroblastic cell lines were characterized by RT-qPCR, western blot and immunofluorescence assay. Our results showed that nF and mF cells were positive for fibroblastic markers such as vimentin and collagen 1, and negative for cytokeratin 18 and E-cadherin, attesting to their fibroblastic type. They also expressed proteins characteristic of the epithelial stem cell niche such as Grem1, CD90 or Wnt5a. Only rare nF fibroblasts were positive for α-SMA, whereas all mF fibroblasts strongly expressed this marker, supporting that mF cells were activated fibroblasts/myofibroblasts. In coculture, nF fibroblasts and Co cells strongly interacted *via* paracrine exchanges resulting in BMP4 production in nF fibroblasts, activation of BMP signaling in Co colonocytes, and decreased growth of colonocytes. Activated-type mF fibroblasts did not exert the same effects on Co cells, allowing colonocytes free to proliferate. In conclusion, these two colonic fibroblast lines, associated with Co cells in coculture, should allow to better understand the role of mesenchymal cells in the preservation of homeostasis and the development of intestinal pathologies.

## Introduction

The colonic epithelium, a monolayer of polarized cells organized into crypt units, is located at the interface between the internal milieu and the hostile luminal environment (luminal pathogenic microorganisms, pro-inflammatory molecules, bile salts and other deleterious luminal factors). Accordingly, the colonic epithelium constitutes the most fundamental element of the protective colonic barrier and any disruption of this cell monolayer can lead to the loss of barrier integrity and then promote or induce the development of intestinal pathologies such as chronic inflammatory bowel disease (IBD) or colorectal carcinoma (CRC) [1, 2]. In order to maintain an efficient barrier function and colonic integrity, the epithelium is in constant and rapid renewal (every 4–5 days in humans) through leucine-rich repeat-containing G protein-coupled receptor 5 (Lgr5)+ colonic stem cells (CSCs) located at the bottom of the crypt [3]. CSCs produce committed progeny that mature into different types of epithelial cells (colonocytes, endocrine cells, goblet cells and tuft cells) while following a rapid migratory pathway up the crypt where they are removed by detachment-induced apoptosis [4, 5]. This rapid epithelial renewal is a hallmark of intestinal homeostasis and requires a tightly regulated balance between epithelial proliferation, differentiation and apoptosis. To achieve this, CSCs are in continuous interplay with a complex surrounding microenvironment (called niche) which includes subepithelial fibroblasts, lymphocytes, macrophages, endothelial cells and neuronal cells [6]. This microenvironment provides the epithelium, and in particular the CSCs, with a set of contextual information in the form of cytokines, growth factors or signaling cascades such as Wnt, Notch, Hippo and bone morphogenetic protein (BMP) pathways [6]. These subepithelial cells regulate the expression of transcription factors and direct the fate of crypt progeny cells in response to various stimuli, including environmental factors (pro-inflammatory cytokines, dietary factors and neoformed compounds, microbiota residing in the colonic lumen…) [6]. The responses and adaptations of epithelial cells to these factors are critical events for the maintenance of colon homeostasis or the development of intestinal pathologies.

Subepithelial fibroblasts, including myofibroblasts located at the bottom of the crypt, are a major components of the CSCs niche [7]. They form a cellular network close to the epithelium from which they are separated by a very thin layer of extracellular matrix. They communicate in a paracrine fashion with surrounding cells and play a major role in the fine regulation of epithelial proliferation and differentiation along the crypt axis, in the production and remodeling of the extracellular matrix and in the mucosal repair process. Therefore, colon subepithelial fibroblasts are now receiving considerable attention and it seems difficult to overlook their role in the study of epithelial function regulation and homeostasis. Moreover, recent findings have revealed that fibroblasts are critical key players in the pathogenesis of intestinal diseases, including IBD and colonic carcinogenesis. In a pathological environment, fibroblasts acquire an activated form. They are then called activated fibroblasts (AFs), cancer-associated fibroblasts (CAFs) or tumor-associated fibroblasts. Activated type fibroblasts, called myofibroblasts, are also present in the CSCs niche. Among the mechanisms regulating fibroblast activation, many studies have emphasized the importance of the Wnt signaling pathway in the evolution of a resting fibroblast towards an activated fibroblast or towards a myofibroblast [8–11]. AFs can exhibit different phenotypes but the majority of them express α-smooth muscle actin (α-SMA). Several studies have shown that in response to inflammatory signals such as interleukin-1β, tumor necrosis factor-α, or even in response to reactive oxygen species, AFs produce and release various chemokines and cytokines that attract and activate immune cells to sites of inflammation. Therefore, AFs play a pivotal role in facilitating the switch from acute resolving inflammation to chronic inflammation [12–14]. In the context of tumor development, several subtypes of CAFs arise from continuous mutual interaction with neighboring preneoplastic and neoplastic epithelial cells [15]. Some CAFs subpopulations restrain tumor initiation and progression, such as do non-activated fibroblasts, while others promote tumor development, notably by increasing proliferation, survival and invasion. For example, subcutaneous injection of CAFs with human colon cancer HCT116 cells markedly promoted tumor growth in mice as compared with the injection of HCT116 cells alone [16]. Likewise, *in vivo* experiment in SCID (Severe Combined ImmunoDeficiency) mice clearly demonstrated that the injection of colon cancer stem cells with CAFs in the graft enhanced the tumor take [17]. The involvement of CAFs has also been demonstrated in a mouse colitis-associated CRC model [18, 19].

Despite the importance of subepithelial fibroblasts in the preservation of homeostasis but also in the development of various intestinal pathologies, it remains unclear how these cells communicate and interact with the epithelial layer, and especially reflect changes in the microenvironment. The lack of appropriate *in vitro* models, in particular resting and activated colonic fibroblasts, can explain this at least in part. Therefore, the objective of our study was to understand the regulation of epithelial-mesenchymal crosstalk through the establishment of a well-defined *in vitro* co-culture model. To this end, we characterized two novel conditionally immortalized fibroblast cell lines derived from mouse colon, one fibroblast-like cell line and one activated fibroblast-like cell line. The establishment of these lines then allowed us to establish a co-culture system combining these fibroblasts with conditionally immortalized normal mouse colonocytes to understand the influence of the epithelial-mesenchymal crosstalk on the proliferation and behavior of colonocytes according to the fibroblast subtype.

## Material and methods

### Cell lines

The normal wild-type conditionally immortalized colon epithelial cells, here called Co, were established as described previously [20–22]. The two conditionally immortalized fibroblast cell lines, called nF fibroblasts and mF fibroblasts, were prepared at the same time. Briefly, a heterozygous female “Immortomouse” was mated with a male C57BL/6J mouse to obtain normal fibroblasts (nF) or with a heterozygous male Min/+ mouse to obtain fibroblasts mutated for Apc (mF). The transgenic H-2Kb-tsA58 mouse [23] or “Immortomouse” (Charles River Laboratories, Wilmington, MA, USA) has a temperature-sensitive mutation of the simian virus 40 large tumor antigen gene (tsA58) under the control of interferon γ (IFN-γ) which induces the H-2Kb promoter. All the cells of these mice are “immortalized,” i.e., they express the SV40 antigen, which can be turned on by culturing the cells *in vitro* with interferon γ at a permissive temperature (33°C) for the ts-A58 mutation. The progeny F1 were typed using PCR for *Apc* mutation [20–22]. Crypt cells from the colon of F1 mice were cultured under permissive conditions (33°C, interferon γ). *Ap*c+/+ epithelial clones (Co), *Ap*c+/+ fibroblastic clones (nF) and *Ap*c+/*Min* (mF) fibroblastic clones were selected after a clonal dilution and then used for characterization and further studies [20–22]. The selection of fibroblasts derived from a cross with the Apc Min+/- mouse seemed particularly interesting in the present study because this mouse strain carries an autosomal dominant mutation in the gene encoding *Apc* at codon 850, leading to the production of an inactive truncated Apc protein, and subsequently to the activation of the Wnt signaling pathway [24]. This animal model thus offered us the opportunity to obtain an activated fibroblast line.

### Cell culture

Epithelial and fibroblastic cells were maintained in tissue culture flasks at permissive temperature of 33°C in a 5% CO_2_ atmosphere in a humidified incubator and in complete culture medium containing Dulbecco-modified essential medium (DMEM GlutaMAX™) supplemented with 10% fetal calf sera, 1% penicillin/streptomycin, 10 U/ml IFN-γ and 10 U/ml epidermal growth factor, as previously described [20, 21]. The medium was changed three times a week and cells were passaged when they were 70% confluent. All experiments were performed at permissive temperature of 33°C [20, 21].

### Whole genome sequencing and Bioinformatics analysis

#### Whole genome sequencing

Genomic DNA for whole-genome sequencing (WGS) was extracted from nF, mF and Co cells using the GenElute Mammalian Genomic DNA Miniprep Kit as described by the manufacturer (Sigma Aldrich, Saint Quentin Fallavier, France). DNA-seq libraries have been prepared according to Illumina’s protocols using the Illumina TruSeq Nano DNA HT Library Prep Kit (Lexogen, Vienna, Austria). Briefly, DNA was fragmented by sonication, size selection was performed using SPB beads (kit beads) and adaptators were ligated to be sequenced. Library quality was assessed using an Advanced Analytical Fragment Analyzer and libraries were quantified by QPCR using the Kapa Library Quantification Kit. DNA-seq experiments have been performed on an Illumina NovaSeq6000 using a paired-end read length of 2x150 bp with the Illumina NovaSeq6000 Reagent Kits. The raw sequences were deposited at SRA under accession PRJNA800346.

#### Bioinformatics analysis

FastQC was used to check quality and no relevant contamination hit was found after the alignment against E. coli, Yeast and PhiX.Reads mapping and variants calling were performed using Nextflow v20.10.0 and Sarek pipeline v2.6.1. Genome Analysis Toolkit (GATK) best practices were followed as implemented in the Sarek pipeline. In this project, the following steps were performed: aligning the reads with BWA v0.7.17-r1188 against the mouse genome assembly C57BL_6NJ_v1 (GCA_001632555.1), marking duplicate reads (MarkDuplicates), base quality recalibration (BQSR) and calling germline small variants (HaplotypeCaller in GVCF mode) with GATK v4.1.7.0 on merged bam by sample. Finally quality control is performed with MultiQC v1.8. Out of nextflow pipeline the variants were annotated with VEP version 103.1 with assembly C57BL_6NJ_v1. Variant with at list quality of 30 and depth of 5 were kept to further analyze. The genome sequencing of the cells are accessible through the following link: https://www.ncbi.nlm.nih.gov/sra/PRJNA800346.

### *In vitro* experiments

#### Direct coculture assay

To allow interactions between the two cell types, fibroblasts (nF or mF) were seeded at a density of 10^5^ cells per well in 6-well plates (Sarstedt, Numbrecht, Germany) and cultured alone during 24 h. Suspensions of Co colonocytes (1.2 x10^5^ cells per insert) were then seeded onto Thincert™-Tissue culture inserts for six well plates with 0.4 μm pore size (Greiner bio-one France, Courtaboeuf, France), and cultured in the presence or absence of fibroblasts on the bottom of the well. Colonocytes or fibroblasts alone were plated in the control inserts or wells. The cocultures were left for 72 h. The culture media under the inserts (conditioned medium, CM) were then collected, centrifuged at 2000 × g for 10 min to remove any debris or cell, and stored at −80°C until later use. We obtained the following conditioned media: **CM-nF/Co:** conditioned medium resulting from the coculture between nF fibroblasts and Co cells; **CM-mF/Co**: conditioned medium resulting from coculture between mF fibroblasts and Co cells; **CM-nF**: conditioned medium of nF fibroblasts in monoculture; **CM-mF**: conditioned medium of mF fibroblasts in monoculture; **CM-Co**: conditioned medium of Co cells in monoculture. Epithelial cells and fibroblasts were then trypsinized, suspended in culture medium and counted with a luna^TM^ cell counter (Logos biosystem, Villeneuve d’Ascq France), before being centrifuged at 1000 × g for 5 min. Cell pellets were washed two times with PBS and stored at −20°C until western blot analysis.

#### Indirect coculture assay

For preparation of conditioned medium, cells (nF, mF, Co) were cultivated to 60% confluency. Their medium was then refreshed by complete culture medium (33°C). The supernatant (conditioned medium, CM) was collected after 72 h as describe above and stored at −80°C until later use on Co cells. For this, Co colonocytes were plated in 8-chambered slide (Lab-tek® Chamber slide^TM^ system, Nunc, Rochester, USA) or in 6-well plates and treated with CM from nF cells (**CM-nF**), mF cells (**CM-mF**) or Co cells (**CM-Co**) at 6, 24 and 48 h. Co cells in 6-well plates were then trypsinized (t = 72 h), suspended in culture medium and counted with a luna^TM^ cell counter. Co cells in 8-chambered slide were fixed/permeabilized with ice-cold 100% methanol for immunofluorescence (IF) assay.

#### Coculture with K02288, a BMP type I receptor inhibitor

nF or mF fibroblasts were seeded at a density of 10^5^ cells per well in 6-well plates and cultured alone during 24 h. Suspensions of Co cells (1.2 x10^5^ cells per insert) were then seeded onto Thincert™ inserts with 0.4 μm pore size, and cultured in the presence or absence of fibroblasts on the bottom of the well. Treatment with K02288 (Sigma Aldrich; 250–500 nM) was performed at the basolateral pole of the colonocytes at t = 6 h. Control cells were treated with an equal volume of dimethyl sulfoxide (DMSO) vehicle. The medium was replaced and the treatment repeated at 24 h and 48 h. At t = 72 h, Co epithelial cells were trypsinized, suspended in culture medium, and counted with a luna^TM^ cell counter. Experiments were repeated at least four times in triplicate.

#### Treatment with BMP signaling agonist SB4

For experiments with SB4 (2-[[(4-Bromophenyl)methyl]thio]benzoxazole; Sigma Aldrich), Co cells were seeded in 6-well plates with 1.2 x10^5^ cells/well or in 8-chambered slide (Lab-tek® Chamber slide^TM^ system) with 2x10^4^ cells/well in 200 μl/well of complete culture medium. The cells were placed in the incubator at 33°C for 6 h to allow them to settle, and then treated with SB4 (0.1–1 μM). Control cells were treated with an equal volume of DMSO vehicle. The medium was replaced and the treatment repeated at 24 h and 48 h. At t = 72 h, Co cells in 6-well plates were trypsinized, suspended in culture medium, and counted with a luna^TM^ cell counter. A minimum of three counts per well were performed. Experiments were repeated at least four times in triplicate. Cells from 8-chambered slide were fixed for IF assay.

### Immunofluorescence

For the characterization of fibroblasts, cells were seeded at approximately 2x10^4^ cells/well in an 8-chambered slide (Lab-tek® Chamber slide^TM^ system) and allowed to settle for 48 h at which times cells were and fixed/permeabilized with ice-cold 100% methanol for 10 min and washed with ice-cold PBS. Cell preparations were then blocked with 2.5% normal horse serum for 20 min before the first primary antibody (Ab), diluted with Animal-Free Blocker® and Diluent (Vector laboratories, Burlingame, CA, United States), and was applied at room temperature for 2 h. After PBS baths, slides were incubated for 15 min with Amplifier Antibody (VectaFluor™ Excel Amplified Kit, Vector laboratories). After PBS rinse, the cell preparations were incubated for 30 min with VectaFluor Reagent (DyLight®594 or DyLight®488, Vector laboratories). Cell preparations were then counterstained with DAPI (Sigma Aldrich) for 1 min. After being washed, the coverslips were mounted. IF microscopy was performed using the primary Abs listed in S1 Table. The specificity of primary Abs was checked in negative controls that omit the primary antibody only.

For indirect and direct coculture, cells were seeded at approximately 2x10^4^ cells/well in an 8-chambered slide and allowed to settle for 6 h. The media was then removed and replaced with conditioned media to be tested. The CM were renewed at 24 h and 48 h. At 72 h, cells were then fixed/permeabilized with ice-cold 100% methanol for 10 min, washed with ice-cold PBS and treated as previously described. Cellular proteins were stained with one of the following Abs (rabbit anti–Ki67, rabbit anti-active caspase 3, rabbit anti–BMP4, rabbit anti-phospho-Smad1/5, rabbit anti–phospho-Smad4, and rabbit anti-LGR5) (S1 Table). Images were acquired using the Leica Microsystems fluorescence microscope (LEICA DM2000 Led) and Leica DFC7000T camera using a 40x objective. The images were processed using the Leica Advanced Application Suite software (Version 2018.1, Leica Microsystems: Wetzlar, DEU). Five different zones in each well of the culture chamber were counted (with an average of one hundred cells per zone) and an average value was calculated for each cell passage. The experiments were replicated a minimum of four times.

### Real time cell analysis

The method has been extensively described in a previous study [25]. Briefly, a real-time cell analyzer (RTCA iCELLigence™ instrument, ACEA Biosciences, Inc, CA, USA) was used to evaluate the proliferation of fibroblasts. Cell index (CI) impedance measurements were performed according to the instructions of the supplier. For this purpose, cells were re-suspended in media and subsequently adjusted to 10^5^ cells/well or 2.10^5^cells/well in E-plate 8. The instrument was placed in a standard CO_2_ cell culture incubator at 33°C where it transmits data wirelessly to the Control Unit (an iPad housed outside the incubator). The rate of cell growth was determined by calculating the slope of the line between two given time points (0–24 h).

### Cell count assay using luna^TM^ cell counter

For characterization of fibroblasts and colonocytes, cells were trypsinized during the exponential growth phase and suspended in culture medium. The number of cells was then counted (in triplicate) with LUNA^TM^ (Logos Biosystems, Villeneuve d’Ascq, France) using the “Bright-Field Counting mode” as described in the manual. Cell counts and cell size distributions were shown as histograms on the monitor of the LUNA^TM^ cell counter. Before the cells were counted, the upper and lower gates of the counter were adjusted manually to eliminate small particles.

### Protein extraction and western blot analysis

Proteins were extracted in RIPA lysis buffer (0.05 M Tris-HCl, pH 7.4, 0.15M NaCl, 0.25% deoxycholic acid, 1% NP-40, 1 mM EDTA) freshly supplemented with 1X protease inhibitors and 1X phosphatase inhibitors. The cells were extracted with 10 sec of sonication and then on ice for 30 min. The lysates were centrifuged and the supernatants were transferred into new vials. Protein concentration was measured using the BCA assay Pierce method.

Samples were boiled (90°C) for 5 min in classical Laemmli buffer prior to western blotting. Twenty micrograms per lane of total proteins were separated by a gel 10% Mini-PROTEAN® TGX Stain-Free™ (tris glycine) Protein Gels (Biorad, Marne La Coquette, France) at 150 V for about 50min using the Mini-Protean III™ device (Bio-Rad) and then transferred to a Trans-Blot® Turbo Mini 0.2 μm Nitrocellulose Transfer Packs (Bio-Rad) using the Transblot-Blot® SD Semi-Dry Transfer Cell (Bio-Rad) at constant 25V for 7 min.

Membranes were then incubated with primary antibodies overnight (4°C) (S2 Table). Bound antibodies were detected by using the WesternBreeze Immunodetection Chemiluminescent System (Invitrogen, Fisher Scientific, Illkirch, France). The optical density of bands was visualized with the Image System (ChemiDoc, Biorad) and analyzed with Quantity one-image analysis software (Bio-Rad Laboratories).

### Rt-qPCR

Total RNA was isolated from nF or mF cells using the MiniRNeasy Kit (Qiagen, Hilden, Germany). RNA samples (1 μg) were then reverse-transcribed with the iScript™ Reverse Transcription Supermix (Bio-Rad) for real-time quantitative polymerase chain reaction (qPCR) analyses. The primers for Sybr Green assays are presented in S3 Table. Amplifications were performed on a ViiA 7 Real-Time PCR System (Applied Biosystems). The qPCR data were normalized to the level of the Hypoxanthine-Guanine Phosphoribosyltransferase (Hprt1) messenger RNA (mRNA) and analyzed by the LinRegPCR v.11 software.

### Statistical analysis

The results were analyzed using software GraphPad Prism 9.3.0 for Windows. Different responses were analyzed by one-way ANOVA and student’s *t*-test, respectively. When ANOVA showed a statistically significant effect (P<0.05), comparison among data was done using Tukey’s HSD Post-hoc test. Results are expressed as mean ± SEM and P<0.05 was considered significant.

## Results

### Characterization of nF and mF cell lines

#### General characteristics

**Fig 1:**
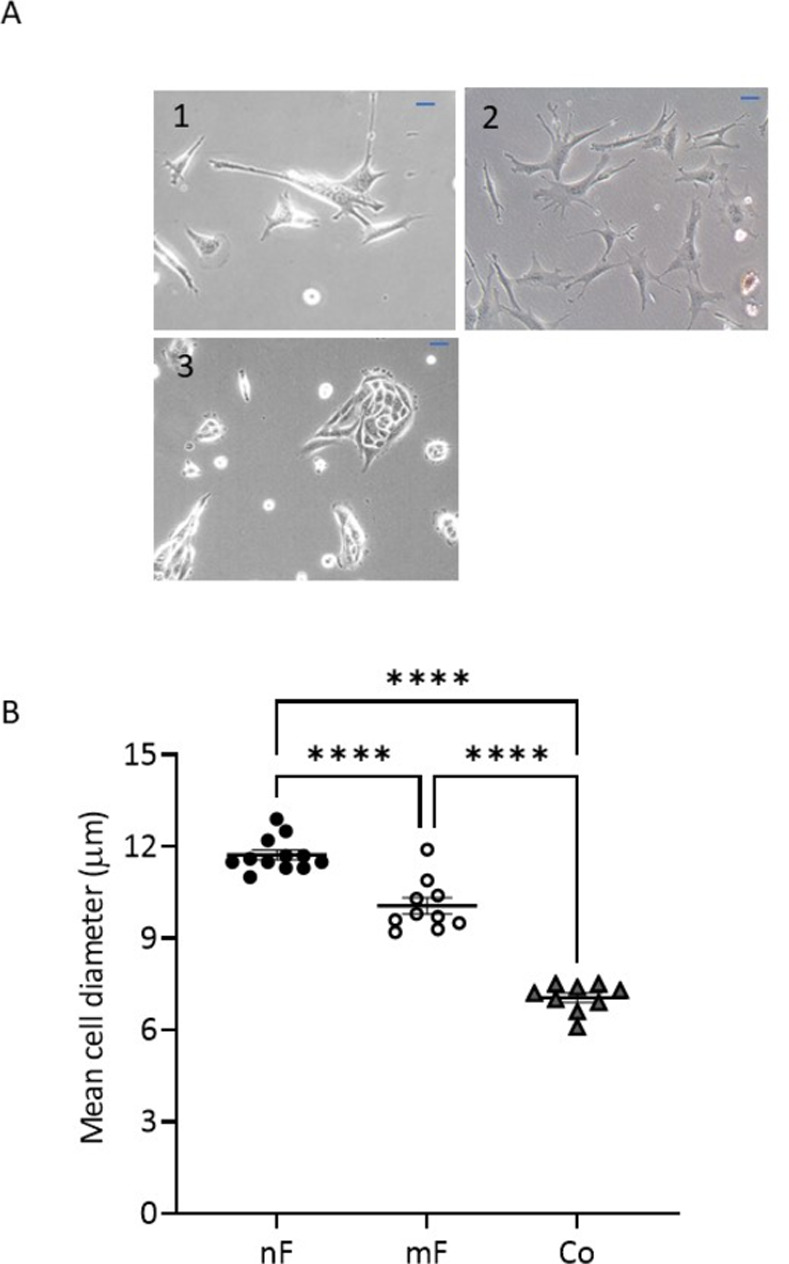
Morphological characteristics of fibroblastic cell lines. A) Phase-contrast microscope images of living fibroblasts and Co cells: (1) nF Fibroblasts, (2) mF Fibroblasts and (3) Co cells. Representative images are shown (Scale: 50 μm). B) Mean diameter of fibroblasts and epithelial cells as measured using Luna^TM^ cell counter after trypsinizing the cells (n≥10 different cell culture passages). Values were presented as means ± SEM. **** P<0.0001.

The nF cells were elongated and spindle-shaped cells, whereas the mF cells were rather stellate or polygonal (Fig 1A). The mean diameter of nF cells was significantly larger than that of mF cells (P<000.1, Fig 1B). In comparison, the epithelial Co cell line had a smaller cell diameter than the other two lines. Epithelial cells and fibroblasts also showed a different cell size distribution (S1A Fig). For fibroblasts, the histogram of cell number versus cell size showed two peaks: one around 5–6 μm and another around 11–12 μm. Using iCELLigence, we showed that the average slope of the growth curve between 0–24 h was 0.34 for mF instead of 0.1 for nF, suggesting that mF cells proliferate faster than nF cells (S1B Fig). Whole genome sequencing and bioinformatics analysis confirmed the presence of the *Apc* mutation in the mF fibroblast cell line (S2 Fig), whereas this mutation was absent in nF fibroblasts and Co cells. The mutation corresponded to the *Apc* Min/+ phenotype with a stop codon at position 850. As result, most of the Apc proteins produced by mF cells were truncated (S2 Fig). We also showed that all three cell lines did not have mutations in *TP53*, *KRAS*, *SMAD4* and *BRAF* (SRA files accessible through the following link: https://www.ncbi.nlm.nih.gov/sra/PRJNA800346).

#### Fibroblastic markers

**Fig 2:**
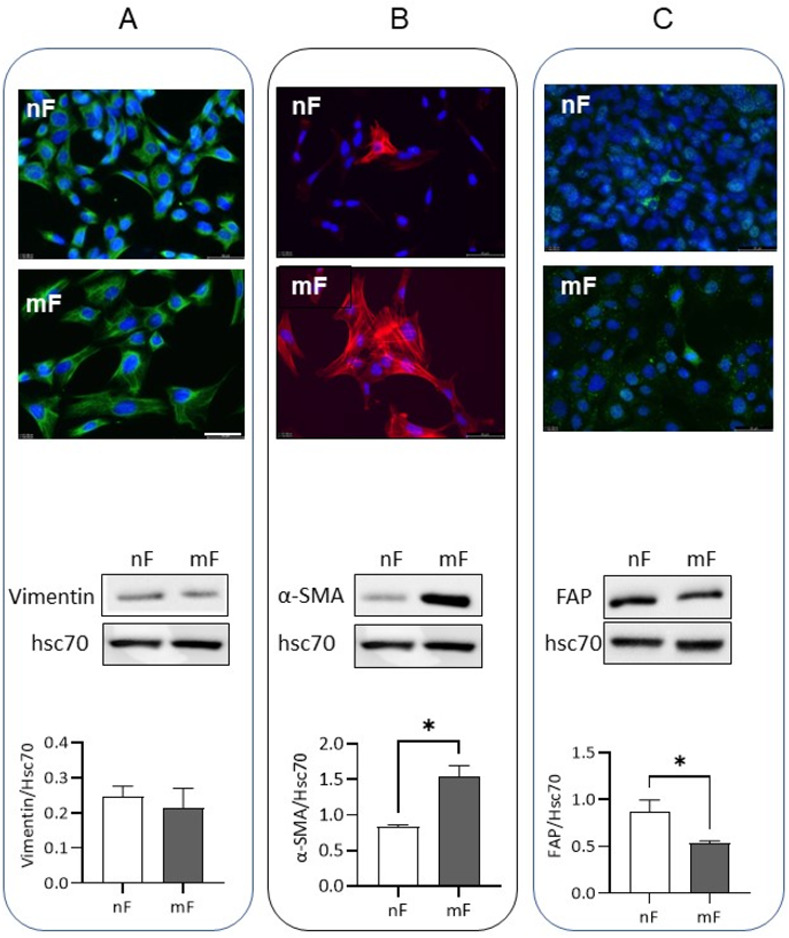
Fibroblast markers determined by IF and western blot. nF and mF fibroblasts were evaluated for the expression of vimentin (A), α-SMA (B) and FAP (C). For IF assay, the pictures were representative of four separate experiments. Alexa Fluor 488 goat anti-rabbit or 594 goat anti-rabbit were used for secondary antibody and DAPI staining for nucleus. Scale bar shows 50 μm. For western blot analysis, levels were normalized to Hsc70 and data were expressed as mean ± SEM of 3 different cell passages. *P<0.05.

**Fig 3:**
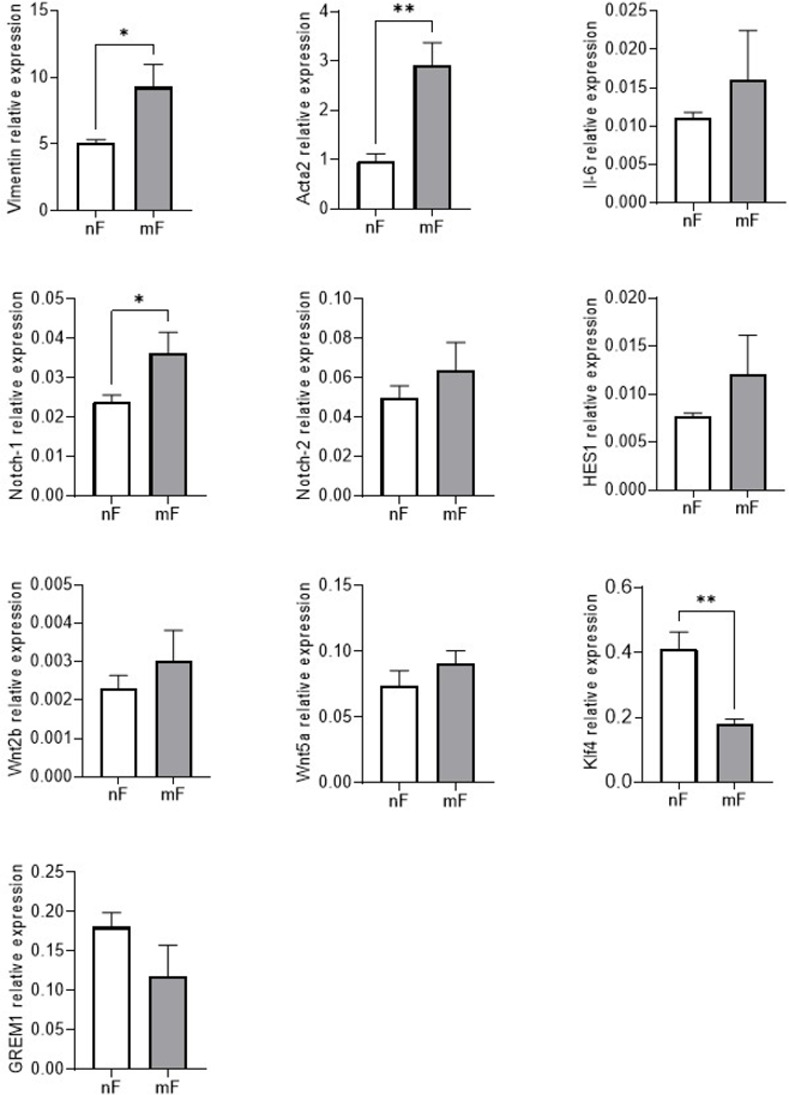
Relative mRNA expression of *vimentin*, *Acta2*, *Notch1*, *Notch2*, *HES1*, *Klf4*, *Wnt2b*, *Wnt5a*, *Il-6* and *Grem1* in nF and mF fibroblasts. The qPCR data were normalized to the level of the Hypoxanthine-Guanine Phosphoribosyltransferase (*Hprt1*) messenger RNA (mRNA) and analyzed by the LinRegPCR v.11 software. Data were expressed as mean ± SEM (n = 4). * P < 0.05; ** P<0.01.

IF assays showed that all cells in both fibroblast populations expressed the fibroblast marker vimentin (Fig 2A) and were negative for cytokeratin 18 and E-cadherin (S3 Fig), indicating that they were prepared with minimal contamination by epithelial cells. Western blot data indicated no significant difference in vimentin expression between nF and mF fibroblasts. In contrast, Co cells strongly expressed cytokeratin 18 and E-cadherin (S3 Fig). With regard to α-SMA, only a few nF fibroblasts showed IF labelling, whereas all mF cells were strongly positive for this marker (Fig 2B). The expression pattern remained constant for more than 6 passages. Western blotting (Fig 2B) and reverse transcription qPCR (Fig 3) confirmed the overexpression of α-SMA/*ACTA2* in mF fibroblasts compared to nF fibroblasts, indicating an activated fibroblast status. In contrast, higher protein expression of FAP was observed in nF than in mF fibroblasts (P<0.05, Fig 2C). Type I collagen (COL1A), which is a major component of extracellular matrix proteins, is composed of two α1 chains and one α2 chain, encoded by *COL1A1* and *COL1A2*, respectively. RT-qPCR as well as IF analysis using an antibody against COL1A1 demonstrated that the two cell lines expressed this marker (S4 Fig). A higher expression of *COL1A2* mRNA was observed in mF fibroblasts compared to nF cells (S4 Fig). CD90/Thy-1, a marker for fibroblasts in the CSC niche [7], was detected in almost all nF cells whereas very few mF cells were labeled (S5 Fig).

As the Notch signaling cascade is involved in fibroblast activation, we also used RT-qPCR to analyze the expression of Notch receptors (*Notch1* and *Notch2*) and their target gene *Hairy and enhancer of split 1* (*Hes1*). Fig 3 showed higher *Notch1* receptor expression in mF fibroblasts than in nF fibroblasts (P<0.05). The mRNA level of *Hes1* also tended to be higher in this cell line (p>0.05). In contrast, the transcription factor Krüppel-like factor 4 (*Klf4*) mRNA level was lower in mF fibroblasts than in nF fibroblasts (P<0.05, Fig 3).

#### Soluble signals of the stem cell niche/stem cell growth factor

RT-qPCR data demonstrated that both fibroblast lines expressed *Il-6*, *Wnt2b* and *Wnt5a* (two canonical ligands of the Wnt pathway), as well as *Gremlin1* (*GREM*1) (a BMP pathway antagonist), with no difference between the two cell lines (Fig 3). As shown in S5 Fig, R-spondin 1 (Rspo1) and R-spondin 3 (Rspo3) proteins were present in nF and mF fibroblasts, with an IF labelling present in all observed cells. We also noted that only rare nF and mF fibroblasts were positive for BMP4.

### Impact of fibroblasts on epithelial cells in coculture

#### nF Fibroblasts but not mF fibroblasts reduced epithelial cell growth

**Fig 4:**
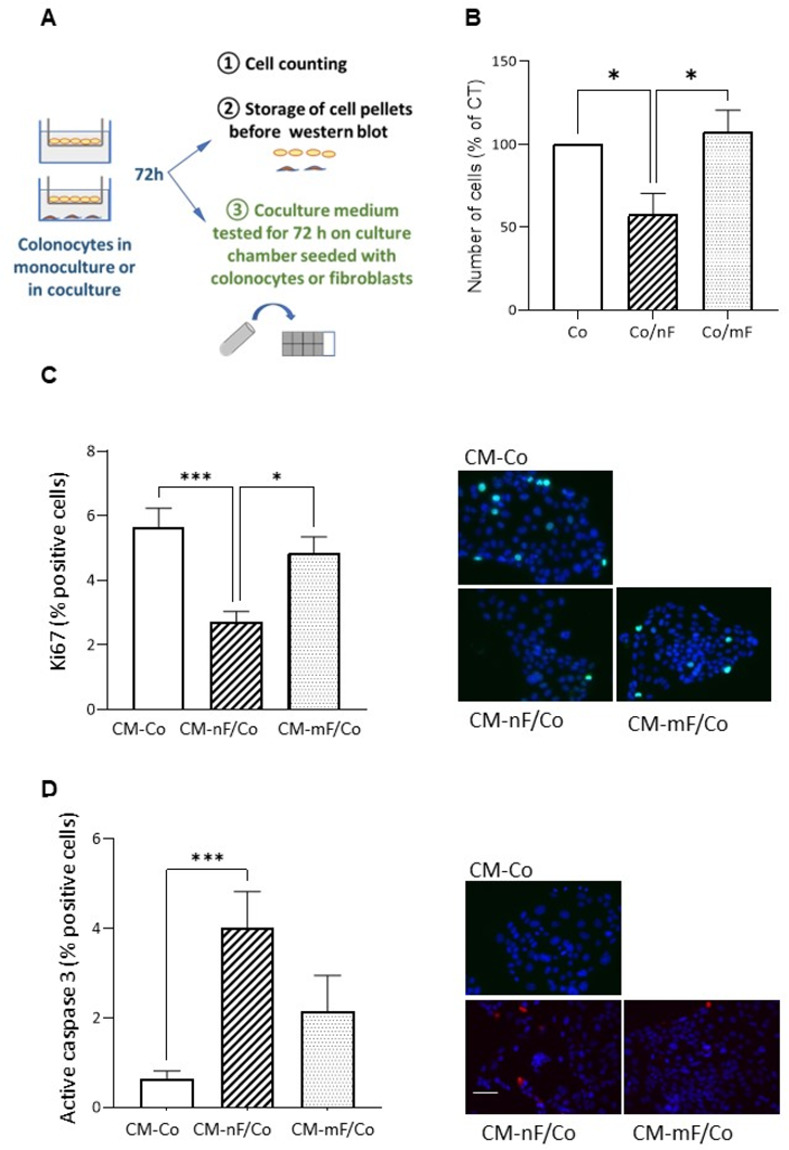
Effects of colonic fibroblasts on Co epithelial cells in direct coculture system. A) Diagram describing the direct coculture protocol as well as the use of the conditioned medium collected after 72 hours of coculture. B) Effect on the number of Co cells. Co cells cultured alone (without fibroblasts in co-culture) were defined as a control. The graphs represented the mean ± SEM from 4 independent experiments. *P<0.05. **Co/nF**: Co cells in direct coculture with nF fibroblasts. **Co/mF**: Co cells in direct coculture with mF fibroblasts. C and D) Effect of CM from direct coculture on proliferation (Ki67) and apoptosis (active caspase 3) of Co colonocytes grown on 8-chambered slides. The number of apoptotic or proliferative cells following treatment with CM for an additional 72 h (n≥3). The ratio of positive cells were calculated. Data were expressed as mean ± SEM. *P<0.05, ***P<0.001. Representative IF images were shown. Scale bar shows 50 μm. **CM-nF/Co:** conditioned medium resulting from the coculture between nF fibroblasts and Co cells; **CM-mF/Co**: conditioned medium resulting from coculture between mF fibroblasts and Co cells; **CM-Co**: conditioned medium of Co cells in monoculture.

A representation of the direct coculture model is shown in Fig 4A. The presence of nF-type fibroblasts decreased the number of Co cells by 40.5% after 72 h of coculture (P<0.05; Fig 4B). In contrast, mF fibroblasts had no effect on the epithelial cell population. In turn, epithelial Co cells induced a decrease in the population of nF fibroblasts, but not of mF fibroblasts (S6A Fig). Of note, conditioned medium (CM) of nF fibroblasts cultured alone had no effect on the number of Co cells (S7 Fig).

As shown in Fig 4C, CM-nF/Co significantly decreased the number of Ki67-positive Co cells (2.7% vs 5.7% in CM-Co, P<0.001) whereas CM-mF/Co did not. The CM from nF fibroblasts cultured alone had no effect on the number of Ki67-positive Co cells (S7C Fig).

The percentage of Ki67-positive nF fibroblasts was about 5.3% when cultured in CM-nF, whereas only 3.0% of nF fibroblasts cells were positive for Ki67 when in CMC-nF/Co (S6B Fig). In contrast, CMC-mF/Co only tended to decrease the number of proliferating mF-type fibroblasts.

We found that very few Co cells cultured in CM-Co medium were positive for active caspase 3, whereas culture in CM-nF/Co medium induced a 4.4-fold increase in the percentage of epithelial cells positive for this marker (P<0.001) (Fig 4D). CM-mF/Co was without significant effect (Fig 4D). The percentage of active caspase 3-positive nF cells was also significantly higher when cultured in CM-nF/Co than in CM-nF (S6C Fig).

#### The crosstalk between Co cells and fibroblasts altered protein expression of cellular markers

**Fig 5:**
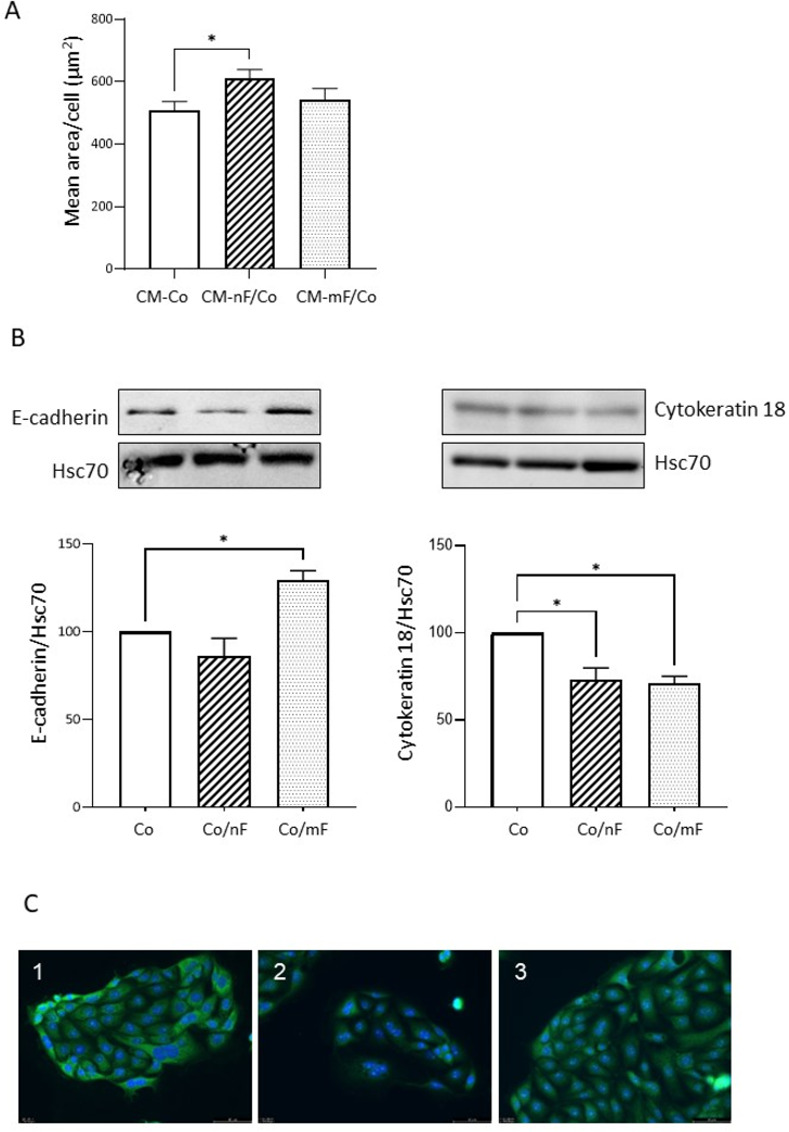
Effect of nF and mF fibroblasts on the phenotype of colonocytes. A) Effect of the different CM collected after 72 h of direct coculture on the mean area per cell for Co colonocytes growth on 8-chambered slides. *P<0.05. Data were expressed as mean ± SEM of 4 experiments. B) Western blot analysis of E-cadherin and cytokeratin 18 protein expression in Co colonocytes after 72 h of coculture with nF or mF fibroblasts. Results were normalized to Hsc70 and data were expressed as mean ± SEM of 3 experiments. *P< 0.05. C) Representative image of Lgr5 localization (green) in Co colonocytes growth on 8-chambered slides with CM collected after 72 h of direct coculture. The pictures were representative of three separate experiments. Alexa Fluor 488 goat anti-rabbit was used for secondary antibody and DAPI staining for nucleus. 1: CM-Co; 2 CM-nF/Co; 3 CM-mF/Co. Scale bar shows 50 μm.

After 72 h of culture in CM-nF/Co, Co cells developed a slight hypertrophy, as shown by the increase in the average area per cell (Fig 5A). Protein expression of cytokeratin 18 was reduced in Co cells in co-culture with nF or mF fibroblasts (Fig 5B). In contrast, the protein level of E-cadherin was increased in Co cells co-cultured with mF fibroblasts, but not with nF fibroblasts. Like many SV40-immortalized cells [26], Co colonocytes express CSC markers. In this study, we targeted the stem cell marker Lgr5. IF staining after a 72-hour culture period revealed that Lgr5 expression in Co cells was lower in the CM-nF/Co medium culture condition than in culture with CM-Co or CM-mF/Co medium (Fig 5C). This result appeared to be confirmed by western blot after a 72-hour coculture period (S8 Fig).

Western blot analysis also showed that α-SMA protein expression was significantly increased only in nF cells. For both nF and mF fibroblasts in direct coculture, the presence of Co cells did not alter the protein expression of FAP (S9 Fig).

### BMP4: A paracrine factor in the crosstalk between nF and Co cells

#### BMP4 expression in fibroblasts and receptor-regulated Smad phosphorylation in epithelial cells after exposure to conditioned medium from direct coculture

**Fig 6:**
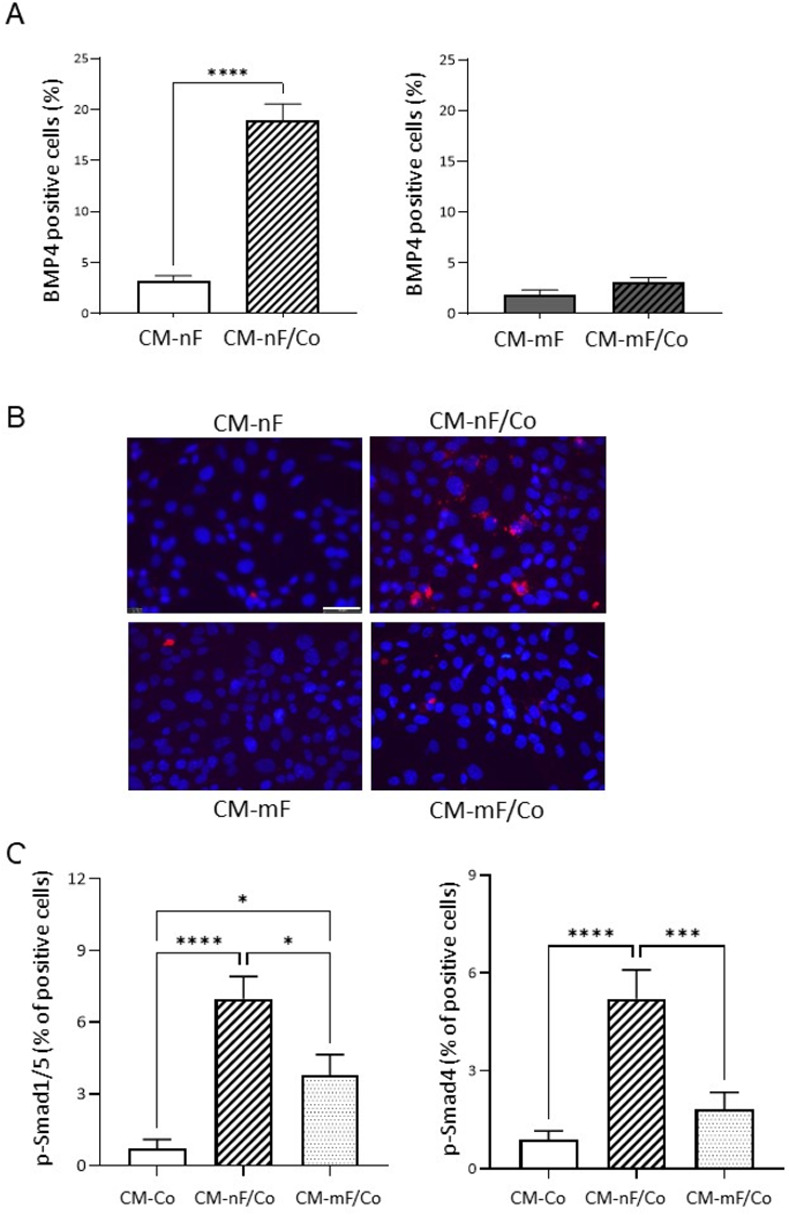
Crosstalk between epithelial Co cells and nF fibroblasts activated the BMP4/Smad pathway. A) Percentage of BMP-4 positive fibroblasts growth on 8-chambered slides with CM collected 72 h after direct coculture. The graphs represented the mean ± SEM values from 4 independent experiments. ***P<0.001. B) Image of IF staining using a BMP4 antibody on fibroblasts. Scale bar shows 50 μm. C) Percentage of p-Smad1/5 and p-Smad4 positive Co colonocytes exposed for 30 min to direct coculture CM in 8-chambered slide. The ratio of positive cells was calculated. Data were expressed as mean ± SEM (n≥3). * P< 0.05; *** P<0.001; **** P<0.0001.

IF analysis of nF fibroblasts revealed an increase in BMP4-positive cells after 72 h of culture in CM-nF/Co medium compared with CM-nF exposure (18.96% *vs* 3.23% positive cells, P<0.001) (Fig 6A and 6B). On the contrary, CM-mF/Co did not induce an increase in BMP4-positive mF fibroblasts. Because Smad1/5 receptor phosphorylation is considered to reflect BMP signaling, we next tested the effect of different coculture CM on the level of p-Smad1/5 in Co cells after 0.5 h of exposure. As shown in Fig 6C, CM-nF/Co increased p-Smad1/5-positive Co cells by a factor of ten compared with CM-Co (P<0.0001). CM-mF/Co also induced a small but significant rise in p-Smad1/5-positive Co cells. The Smad4 co-activator can also be phosphorylated, which plays a central role in regulating its transcriptional activity and stability. Our results showed that CM-nF/Co induced a rise in p-Smad4-positive Co cells, compared with CM-Co or CM-mF/Co media.

#### Effect of a BMP4 antagonist or agonist on epithelial cells

**Fig 7:**
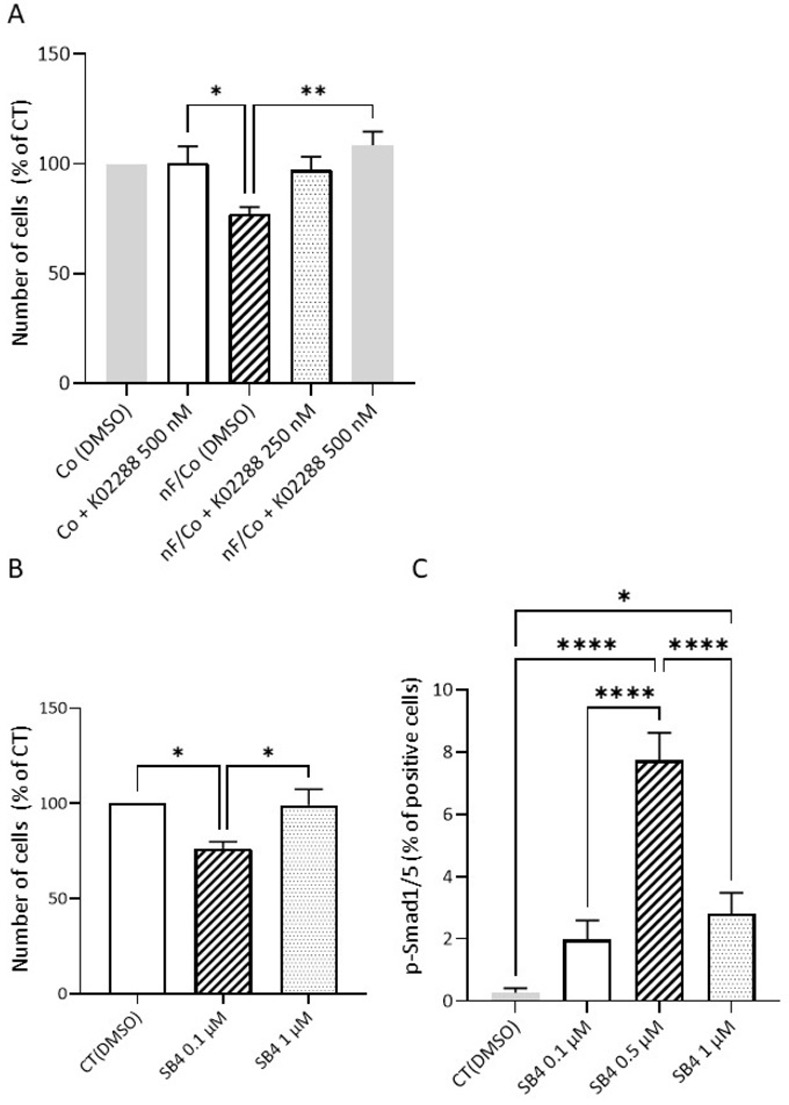
Involvement of the BMP pathway. A) Effect of BMP pathway inhibitor (K02288; 250–500 nM) on nF fibroblast-induced colonic cell growth in direct coculture. Values are mean ± SEM (n = 4 independent experiments). *P<0.05. ** P<0.01. B) Effect of SB4, a BMP4 agonist, on the number of Co cells. The graphs represented the mean ± SEM values from 4 independent experiments. *P<0.05. C) Effects of SB4 on BMP pathway activation in Co cells: Immunostaining of p-Smad1/5 was performed in Co colonocytes exposed to SB4 in 8-chambered slide and the percentage of positive cells was calculated. The graphs represented the mean ± SEM values from 4 independent experiments. * P<0.05; **** P<0.0001.

As shown in Fig 7, inhibition of BMP signaling by the addition of K02288 (500 nM), a potent and selective type I BMP receptor inhibitor, prevented the effect of nF fibroblasts on the reduction of Co cell growth (P<0.001; Fig 7A). Treatment of Co cells with SB4 at 0.1 μM, a BMP4 agonist, significantly decreased cell growth (P<0.05) (Fig 7B). SB4 also increased the percentage of Co cells positive for p-Smad1/5 at all concentrations tested (Fig 7C).

## Discussion

In this study, we investigated the epithelial-mesenchymal interactions between activated or resting fibroblasts and the normal mouse colonocyte cell line through the establishment of two immortalized fibroblast cell lines from mouse colon. Their fibroblastic character was confirmed by their morphology and the expression of markers such as vimentin and collagen I as well as by the absence of protein expression of cytokeratin 18 and E-cadherin. Both fibroblast cell lines also expressed factors essential for epithelial stem cell preservation, such as canonical Wnt pathway ligands (Wnt2b and Wnt5a), Bmp pathway inhibitor (Grem1), agonists of the canonical Wnt/β-catenin signaling pathway (Rspo1 and Rspo3), but also Il-6, BMP4 and CD90 [27–29]. It is important to note that *in vivo*, CD90-positive fibroblasts were identified as belonging to the stem cell niche [7]. They represent a small percentage of colonic fibroblasts, observed mainly in the crypt bottom, close to the stem cells and they express crucial stem cell growth factors such as Grem1, Wnt2b and Rspo3 [7]. Since the majority of nF cells express CD90, this fibroblast cell line could be of great interest to study the mechanisms of regulation of epithelial cell proliferation.

However, these two fibroblast cell lines differ in terms of morphology, phenotypic characteristics and genotypic status. The mF fibroblasts were notably characterized by the presence of a nonsense mutation at codon 850 of the *Apc* coding gene and the subsequent production of a truncated and non-functional Apc protein, which leads to an increase of the Wnt signaling pathway [30]. As Wnt signaling plays an important role in the activation of fibroblasts and their transformation into AFs or CAFs [10, 11], this genotypic characteristic of the mF cells allows to have fibroblasts with a stable activated phenotype, and thus to avoid the activation phase usually achieved with TGF-β1 when using some colonic fibroblastic lines [31–34]. Because of the presence of this mutation, α-SMA expression was much higher in mF fibroblasts, with immunostaining present in all cells, whereas only rare nF fibroblasts were positive for this marker. This result was confirmed by the higher level of *ACTA2* mRNA in mF cells. However, other factors could also be involved in the overexpression of α-SMA in mF cells, in particular Notch1, a member of the Notch receptor family, which was more expressed in mF fibroblasts than in nF fibroblasts. The Notch signaling pathway is known to influence cell status decisions such as survival or apoptosis, proliferation and differentiation, and maintains stem cell quiescence and identity [35]. To date, four Notch receptors (Notch1-4) have been identified in mammals. Among them, Notch1 and Notch3 are known to stimulate α-SMA expression in lung fibroblasts and pancreatic stellate cells [36–38]. Finally, it is interesting to note that Klf4, a negative regulator of fibroblast activation [39], was much more expressed in nF than in mF fibroblasts.

In addition to the greater expression of *ACTA2*/α-SMA, mFs also expressed more *COL1A2*, a hallmark of myofibroblasts [40], than nFs and they also proliferated more rapidly. Taken together, this suggests that mFs are either AFs or CAFs. In agreement with this, Tortora et al demonstrated that *Apc*-mutated colon fibroblasts isolated from apparently-normal tissue colon of 1 month aged Pirc rats (F344/NTac-*Apc*^am1137^), a genetic model of *Apc*-driven CCR, had a constitutive inflammatory and proliferative phenotype, which may promote the establishment of a pro-tumorigenic environment for the development of preneoplastic lesions [41]. Finally, it is important to mention that the two fibroblast lines are themselves heterogeneous, particularly with respect to the expression of BMP4 and FAP, with more intense IF labeling in a few cells of each cell line. As FAP is a marker observed in a subtype of AFs during inflammation, tissue injury and wound healing, as well as during tumor development [42], we can conclude that the mF lineage had different subpopulations of AFs while nF cells contained a small subpopulation of AFs.

What is the value of these two cell lines from a functional point of view? It is now recognized that intestinal fibroblasts play a crucial role in the maintenance of intestinal health, notably by regulating the proliferation and differentiation of the colonic epithelium. However, some activated forms of these mesenchymal cells may also be involved in the progression of intestinal pathologies. Here, we showed that nF fibroblasts in coculture decreased colonocytes growth and repressed expression of the stem cell marker Lgr5 whereas mF fibroblasts did not. Crosstalk between fibroblasts and Co cells also significantly repressed the expression of cytokeratin 8, a type I intermediate filament protein involved in a variety of cellular processes, including cell proliferation, and known to be upregulated in various human tumor tissues [43]. The lack of direct contact between cell types in our coculture model also consistently suggests that nF fibroblasts released soluble factors that, through paracrine interactions, limited epithelial cell proliferation. Multiple signaling pathways have been implicated in the control of CSC proliferation and differentiation, with Wnt signaling constituting the driving force of stem cell proliferation while its main counterforce is represented by ligands of the BMP family [44, 45]. Among the intestinal BMP ligands, BMP4 is mainly expressed in crypt fibroblastic cells while the majority of BMP-generated signals targeted the epithelium [46]. These data support our observation that the dialogue between nF fibroblasts and Co cells resulted in increased BMP4 expression in nF fibroblasts and activation of the BMP pathway in colonocytes, as evidenced by increased Smad1/5 phosphorylation in these cells. We also showed that K02288 prevented the inhibitory effect of the coculture conditioned medium on Co cells growth, signifying the involvement of type 1 BMP receptors. Interestingly, the CM of nF fibroblasts in indirect coculture had no effect on the growth of Co cells, demonstrating the importance of the dialogue between the two cell populations. Together these data suggest that nF fibroblasts controlled epithelial growth *via* BMP4-Smad1/5 signaling following an information exchange with epithelial cells. The mechanism by which Co cells interact with fibroblasts to induce BMP4 production is a part of the dialogue that we have not yet elucidated. It is also important to point out that mF fibroblasts did not respond to this signal given by epithelial cells, leaving the colonocytes free to proliferate, which is an advantage in the case of epithelial injury requiring rapid repair. Indeed, studies demonstrated that loss of BMP response accelerates crypt regeneration to maintain intestinal homeostasis [45]. But this could turn into a problematic situation if fibroblasts do not return to a quiescent state. Consistent with this, aberrant activation of Wnt signaling and loss of BMP signaling have been shown to represent the two major alterations leading to CRC initiation [45]. Our co-culture model and the association between theses cell lines are therefore well functional and reflect the regulations observed *in vivo*.

The question then arises as to whether the mF lineage is indeed representative of AFs or CAFs. Wnt pathway activation, which is here subsequent to the presence of the *Apc* mutation in mF cells, represents one of the main pathways of fibroblast activation [10, 11]. In the colon mucosa, this activation can occur under various pathophysiological circumstances. This is particularly the case in the environment of pre-neoplastic and neoplastic colonic cells, the latter being characterized by an increased production of Wnt ligands in the vicinity of subepithelial fibroblasts [47]. Ferrer-Mayorga et al thus demonstrated that Wnt3a, a ligand highly expressed in CRC, enhanced the expression of the marker *ACTA2*/α-SMA in human colon fibroblasts [48]. Other studies have revealed that epigenetic changes involving hypermethylation of the Wnt antagonists DKK1 and SFRP1 occurring in fibroblasts can lead to activation of the Wnt pathway and thus result in a persistently activated fibroblast status [11]. Finally, patients with familial adenomatous polyposis (FAP), a CRC predisposition syndrome caused by germline mutations in the *APC* gene, rapidly develop large numbers of polyps and the average age of cancer onset in untreated patients is 40 years. Importantly, these patients have mutations in both epithelial and mesenchymal cells and normal colon-derived fibroblasts from a FAP patient were even observed to undergo spontaneous immortalization [49]. mF fibroblasts are therefore representative of FAP colonic fibroblasts and could help to dissect early events leading to CRC development in this syndrome. They will also be of considerable interest to experimentally determine the pathways leading to CRC development and to identify potential molecular targets for cancer prevention. Indeed, although CCR develops from crypt epithelial cells, the proliferation regulation of these cells and the microenvironment modification induced by fibroblasts may play an important role in disease progression [50]. All these data allow us to conclude that the mF lineage is representative of activated fibroblastic cells that could be encountered in pathological situations and is therefore of major interest in coculture systems aiming to determine the role of mesenchyme in the development of intestinal pathologies.

What are the specific limitations and advantages of our cell model? Our co-culture model has some limitations. First, the disadvantage of the strategy used to create our conditionally immortalized colon cell lines is the need to culture the cells at 33°C, which is not a physiological temperature (between 36.5°C and 38°C for mice). Indeed, the SV40 large T gene, an immortalizing gene, carries a temperature-sensitive mutation in our cells [21, 51]. At the permissive temperature of 33°C, the SV40 large T protein is in a conformation that allows it to bind to p53 and inhibit its role in senescence, thus facilitating cell immortalization. By contrast, at the non-permissive temperature of 37°C, the "SV40 large T" protein changes conformation and no longer binds to p53. As a result, the cells die rapidly and do not differentiate. Thus, our model allows a thorough study of the role of fibroblast-epithelial cell interactions on cell proliferation, but is not suitable for studying epithelial differentiation or the epithelial barrier. Other limitations of the model are the absence of cell-matrix interactions and a tissue architecture that is not fully recreated. Therefore, we will only be able to reveal the simple interactions of complex biological processes that occur in tissues in vivo. But on the other hand, the model has some advantages. First, it allows us to work with normal epithelial cells and fibroblasts from adult animals whereas most of the intestinal epithelial cells used *in vitro* are derived either from neonatal rat intestines or from colon carcinomas. Second, mF fibroblasts and Co colonocytes are derived from the same animals. The mF fibroblasts were also obtained from mouse colon and have a genetic background close to Co cells, which is the other major interest of our model. Indeed, the co-culture systems classically used combine cell types from different animals, even from different organs or different species. Our co-culture model, which associates colonocytes and fibroblasts from the mouse colon, allows to reproduce the cellular environment at the base of the colonocytes. Moreover, the model can easily be complexified by adding other types of cells (immune cells, nerve cells). It should facilitate the study of certain mechanisms that occur *in vivo* in experiments on mice (C57BL/6J mice).

## Conclusion

We characterized two fibroblastic cell lines, nF-type and mF-type fibroblasts, the latter resembling so-called activated fibroblasts. Both populations express crucial markers of the stem cell niche such as Grem1, Wnt2b and Wnt5a. We showed that the paracrine interaction between nF fibroblasts and colonocytes involved the BMP pathway and dictated the proliferative activity of epithelial cells, whereas activated mF fibroblasts were without effect on the growth and proliferation of these cells. Although our *in vitro* model has some limitations, particularly related to the absence of the three-dimensional architecture of the colonic stem cell niche, it should nevertheless provide valuable information on the bidirectional communications between colonic fibroblasts and epithelial cells. It will also allow us to investigate and study the secreted factors and receptors that mediate the dialogue between these two cell populations. Such a co-culture model should in the future allow us to gain information on the interplay between epithelial cells and fibroblasts, to better understand the role of fibroblasts in the development of intestinal pathologies and to determine if these cells could have a triggering or aggravating role.

## Supporting information

S1 Table List of antibodies for immunofluorescence. (DOCX)

S2 Table List of antibodies for western blot. (DOCX)

S3 Table Primer sequences for the quantitative PCR. (DOCX)

S1 Fig nF and mF fibroblasts. A) Histogram of cell size distribution: Cell distribution, measured using the electronic Coulter Counter (Luna^TM^) in terms of cell number as a function of cell diameter (μm). Representative example of distribution of cell sizes in colonocytes and fibroblast cell cultures. B) Cell proliferation rate of nF and mF fibroblasts seeded at different seeding concentrations (0.1 or 0.2 million cells) determined by slope analysis between 0 and 24 hours using the iCELLigence system. Experiments were repeated several times (≥ 3), and the results were expressed as mean +/- SEM. * P<0.05.
(TIF)

S2 Fig Genotypic status of mF fibroblasts. A) Alignment of one base substitution in murine mF fibroblasts (fibro-apc-mute track) versus murine nF fibroblasts (fibro-normal track) by the Integrative Genomics Viewer (IGV) and graphic report showed detection of the mutation. B) VEP analysis and Web results (summary pie charts with statistics and results table) of base substitution in murine mF fibroblasts. Only mutations (divided in categories based on their predicted impact on protein functions using snpEff (Cingolani et al, 2012) and classified ‘‘high” (“The variant was assumed to have disruptive impact in the protein, probably causing protein truncation, loss of function or triggering nonsense mediated decay”) were considered. Alignment of one base substitution in murine mF fibroblasts (fibro-apc-mute track) versus murine nF fibroblasts (fibro-normal track) by the Integrative Genomics Viewer (IGV) and graphic report shows detection of the mutation. C) Western blot analysis showing the truncated APC protein (~100 kDa) in mF fibroblasts, nF fibroblasts, Co cells and in Min/+ mouse intestine. Cingolani P., Platts A., Wang LL., Coon M., Nguyen T., Wang, L., Land SJ., Lu X. and Ruden DM. (2012). A program for annotating and predicting the effects of single nucleotide polymorphisms, SnpEff. Fly (Austin) 6, 80–92.
(TIF)

S3 Fig Cytokeratin 18 and E-cadherin expression in Co cells and in fibroblasts. (A) Representative image of cytokeratin 18 expression (green) in Co colonocytes. No labeling was detected in nF and mF fibroblasts. The pictures were representative of three separate experiments. Alexa Fluor 488 goat anti-rabbit was used for secondary antibody and DAPI staining for nucleus. (B) Western blot analysis showing cytokeratin 18 (~48 kDa) in Co cells but not in nF and mF fibroblasts. (C) Representative image of E-cadherin expression (red) in Co colonocytes. No labeling was detected in nF and mF fibroblasts. The pictures were representative of three separate experiments. Alexa Fluor 594 goat anti-rabbit was used for secondary antibody and DAPI staining for nucleus.
(TIF)

S4 Fig Type I collagen expression in nF and mF fibroblasts. (A) Representative image of Col1A1 collagen localization (red) in nF and mF fibroblasts. The pictures were representative of three separate experiments. Alexa Fluor 594 goat anti-rabbit was used for secondary antibody and DAPI staining for nucleus. (B) Relative mRNA expression of *Col1A1* and *Col1A2* in the two fibroblast cell lines. The qPCR data were normalized to the level of the Hypoxanthine-Guanine Phosphoribosyltransferase (*Hprt1*) messenger RNA (mRNA) and analyzed by the LinRegPCR v.11 software. Data are expressed as means ± SEM. *P<0.05.
(TIF)

S5 Fig Markers of the stem cell niche in nF and mF fibroblasts. Representative image of CD90, Rspo1, Rspo3 and BMP4 localization in nF and mF fibroblasts. The pictures were representative of three separate experiments. Alexa Fluor 488 goat anti-rabbit or 594 goat anti-rabbit were used for secondary antibody and DAPI staining for nucleus. Scale bar shows 50 μm.
(TIF)

S6 Fig Effects of Co colonocytes on nF and mF fibroblasts in coculture system. nF or mF fibroblasts were seeded into the bottom of 6-well culture plates. Epithelial cells were cultured on a membrane insert for 72 h with nF or mF fibroblasts. A) Effect on the number of nF and mF fibroblasts. nF or mF Fibroblastic cells cultured alone (without Co cells in coculture) were defined as a control. The graphs represent the mean ± SEM values from 4 independent experiments. ***P<0.001. B and C) The effect of conditioned medium from direct coculture on proliferation (Ki67) and apoptosis (active caspase 3) of nF and mF fibroblasts grown on 8-chambered slides. The number of apoptotic or proliferative cells was evaluated following treatment with conditioned medium for 72 h hours. The ratio of positive cells were calculated respectively (n = 4). **P< 0.01, ****P<0.0001. **CM-nF/Co:** conditioned medium resulting from the coculture between nF fibroblasts and Co cells; **CM-mF/Co:** conditioned medium resulting from coculture between mF fibroblasts and Co cells; **CM-nF**: conditioned medium of nF fibroblasts in monoculture; **CM-mF**: conditioned medium of mF fibroblasts in monoculture.
(TIF)

S7 Fig Effects of colonic fibroblasts on Co cells in indirect coculture system. (A) Diagram describing the indirect coculture model. Co cells were grown alone in conditioned medium from fibroblasts or Co cells in monoculture. The graphs represent the mean ± SEM values from 4 independent experiments. (B) Effect of indirect coculture on the number of Co cells. (C and D) Effect of indirect coculture on active caspase 3 and Ki67 positive Co cells. Co cells were seeded in an 8-chamber slide and allowed to settle for 6 h. The medium was then removed and replaced by the conditioned media to be tested or by fresh medium. The different media were renewed at 24 and 48 h. **CM-Co**: conditioned medium from Co cells in monoculture. **CM-nF**: conditioned medium from nF cells in monoculture. **CM-mF**: conditioned medium from mF cells in monoculture. **M33:** Fresh medium.
(TIF)

S8 Fig Effect of nF and mF fibroblasts on Lgr5 expression in Co cells. The relative protein expression levels of Lgr5 by western blot in Co colonocytes after 72h of direct coculture with nF or mF fibroblasts. Results were normalized to Hsc70 and data were expressed as mean ± SEM (n≥3) *P < 0.05.
(TIF)

S9 Fig Expression of fibroblastic markers in nF and mF cells growth in direct coculture with Co colonocytes. Western blot analysis of α-SMA and FAP protein expression in fibroblasts after 72 h of coculture with Co colonocytes. (A) nF fibroblasts and (B) mF fibroblasts. Results were normalized to HSC70 and data were expressed as mean ± SEM of 4 experiments. *P< 0.05.
(TIF)

S1 Raw images (PDF)
