# Supplementary material for: Study of the colonic epithelial-mesenchymal dialogue through establishment of two activated or not mesenchymal cell lines: Activated and resting ones differentially modulate colonocytes in co-culture
Source: PLoS One. 2022 Aug 30;17(8):e0273858. doi: 10.1371/journal.pone.0273858 (PMC9426876; doi:10.1371/journal.pone.0273858)
Supplement: S1 Table — (DOCX) [file pone.0273858.s001.docx]

**S1 Table. list of antibodies for immunofluorescence.**

| **Target** | **Clonality** | **specy** | **clone** | **Used dilution** |
| --- | --- | --- | --- | --- |
| Vimentin | Polyclonal | Rabbit | A11952 | 1 :200 |
| Cytokeratin 18 | Polyclonal | Rabbit | GTX105624 | 1 :100 |
| E-cadherin | Polyclonal | Rabbit | GTX100443 | 1 :100 |
| FAP | Polyclonal | Rabbit | PA5-51057 | 1 :100 |
| α-Smooth Muscle Actin | Monoclonal | Rabbit | 19245S | 1 :100 |
| Cleaved Caspase-3 | Monoclonal | Rabbit | ≠9664 | 1 :200 |
| Rspo1 | Polyclonal | Rabbit | PAM171Mu01 | 1 :50 |
| Rspo3 | Polyclonal | Rabbit | PA5-38052 | 1 :50 |
| Collagen 1 (Col1A1) | Polyclonal | Rabbit | PA5-95137 | 1 :250 |
| CD90 | Polyclonal | Rabbit | GTX130072 | 1 :250 |
| Ki67 | Monoclonal | Rabbit | ab16667 | 1 :250 |
| Lgr5 | Polyclonal | Rabbit | PA5-87974 | 1 :50 |
| Phospho-Smad4 (Thr277) | Polyclonal | Rabbit | PA5106038 | 1 :100 |
| Phospho-Smad1/5 (ser463/465) | Monoclonal | Rabbit | 9516 | 1 :800 |
| BMP4 | Polyclonal | Rabbit | SAB2700755 | 1 :300 |
