# Supplementary material for: Study of the colonic epithelial-mesenchymal dialogue through establishment of two activated or not mesenchymal cell lines: Activated and resting ones differentially modulate colonocytes in co-culture
Source: PLoS One. 2022 Aug 30;17(8):e0273858. doi: 10.1371/journal.pone.0273858 (PMC9426876; doi:10.1371/journal.pone.0273858)
Supplement: S2 Table — (DOCX) [file pone.0273858.s002.docx]

**S2 Table: list of antibodies for western blot.**

| **Target** | **Clonality** | **specy** | **clone** | **Used dilution** |
| --- | --- | --- | --- | --- |
| Vimentin | Polyclonal | Rabbit | A11952 | 1 :500 |
| Cytokeratin 18 | Polyclonal | Rabbit | GTX105624 | 1 :1000 |
| E-cadherin | Polyclonal | Rabbit | GTX100443 | 1 :1000 |
| FAP | Polyclonal | Rabbit | PA5-51057 | 1 :500 |
| Alpha-Smooth Muscle Actin (D4K9N) | Monoclonal | Rabbit | 19245S | 1 :1000 |
| Collagen 1 (Col1A1) | Polyclonal | Rabbit | PA5-95137 | 1 :1000 |
| Lgr5 | Polyclonal | Rabbit | PA5-87974 | 1 :1000 |
