# Supplementary material for: Study of the colonic epithelial-mesenchymal dialogue through establishment of two activated or not mesenchymal cell lines: Activated and resting ones differentially modulate colonocytes in co-culture
Source: PLoS One. 2022 Aug 30;17(8):e0273858. doi: 10.1371/journal.pone.0273858 (PMC9426876; doi:10.1371/journal.pone.0273858)
Supplement: S3 Table — (DOCX) [file pone.0273858.s003.docx]

**S3 Table. Primer sequences for the quantitative PCR.**

| **Gene** | **Alias** | **sequence primer Forward** | **sequence primer Reverse** |  |
| --- | --- | --- | --- | --- |
|  |  |  |  |  |
| Notch1 | Notch1 | GTGCTCAGTGTGTCCTGTGAG | ACGTCAATGCCTCGCTTCTG |  |
| Notch2 | Notch2 | GTTACCTACCACAACGGCACA | CCAAGAAGCCCTCTGGACATC |  |
| Hes1 | Hes1 | TAAGAAAGATAGCTCCCGGCATTC | CCAGAATGTCTGCCTTCTCTAGC |  |
| Il6 | Interleukin 6 | GTAGCTATGGTACTCCAGAAGAC | ACGATGATGCACTTGCAGAA |  |
| Col1a1 | Collagen 1a1 | TGATGGACCTGCTGGCTCT | ACCACGTTGTCCAGCAATACC |  |
| Col1a2 | Collagen 1a2 | TGCAATCGGGATCAGTACGAAAG | TCCACGTGGTCCTCTGTCTC |  |
| Grem1 | Gremlin 1 | CCCACGGAAGTGACAGAATGAA | GCAACGCTCCCACAGTGTAT |  |
| Vim | Vimentin | CGAGAGAAATTGCAGGAGGAGAT | GTGCTTTCGGCTTCCTCTCTC |  |
| Wnt2b | Wnt13 | GCACGGCTGTTCGGAGATT | ACTCACACCGTGACACTTGC |  |
| Wnt5a | Wnt5a | CACAGTGGACAATACTTCTGTCTTTG | CGTCTCTCGGCTGCCTATTTG |  |
| Acta2 | Actin Alpha 2 | TGACCCAGATTATGTTTGAGACCTT | GGGACAGCACAGCCTGAATAG |  |
| Klf4 | Epithelial Zinc Finger Protein EZF | TCCTTTCCTGCCAGACCAGAT | CATGAGCTCTTGATAATGGAGAGAGG |  |
