# Supplementary figures and images for: Study of the colonic epithelial-mesenchymal dialogue through establishment of two activated or not mesenchymal cell lines: Activated and resting ones differentially modulate colonocytes in co-culture
Source: PLoS One. 2022 Aug 30;17(8):e0273858. doi: 10.1371/journal.pone.0273858 (PMC9426876; doi:10.1371/journal.pone.0273858)

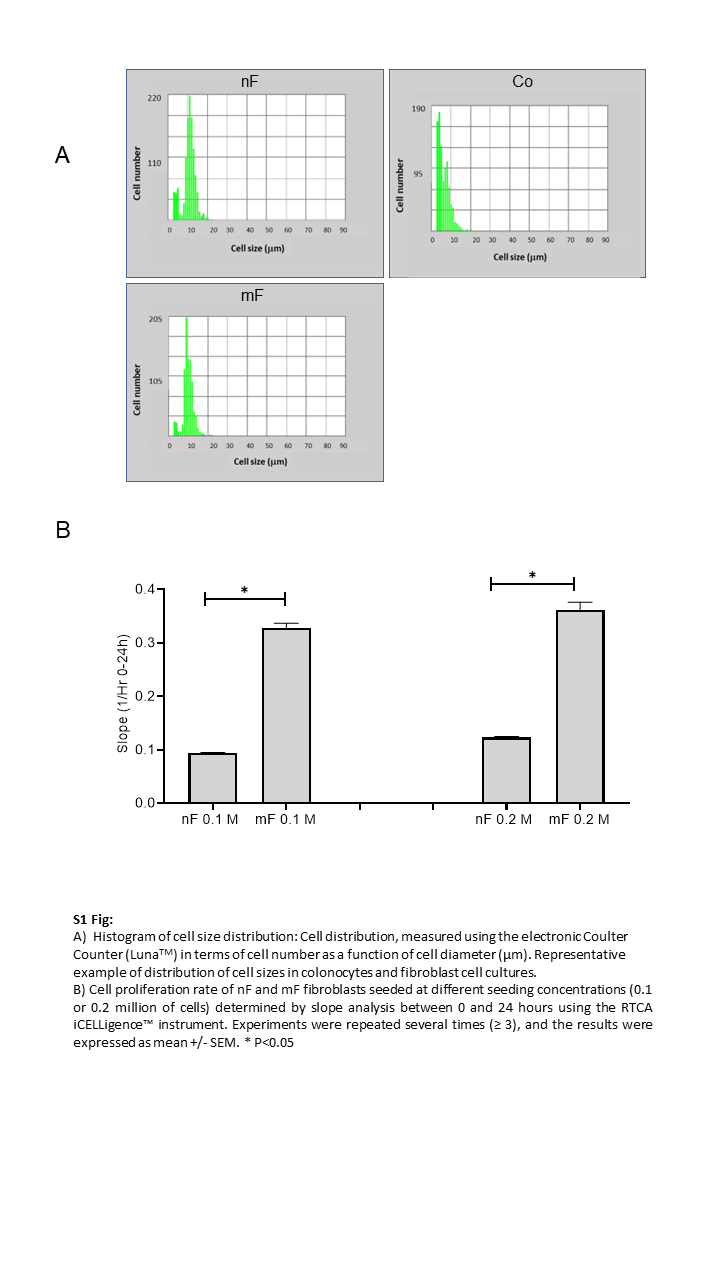

Supplement: S1 Fig — A) Histogram of cell size distribution: Cell distribution, measured using the electronic Coulter Counter (LunaTM) in terms of cell number as a function of cell diameter (μm). Representative example of distribution of cell sizes in colonocytes and fibroblast cell cultures. B) Cell proliferation rate of nF and mF fibroblasts seeded at different seeding concentrations (0.1 or 0.2 million cells) determined by slope analysis between 0 and 24 hours using the iCELLigence system. Experiments were repeated several times (≥ 3), and the results were expressed as mean +/- SEM. * P<0.05. (TIF) [file pone.0273858.s004.tif]

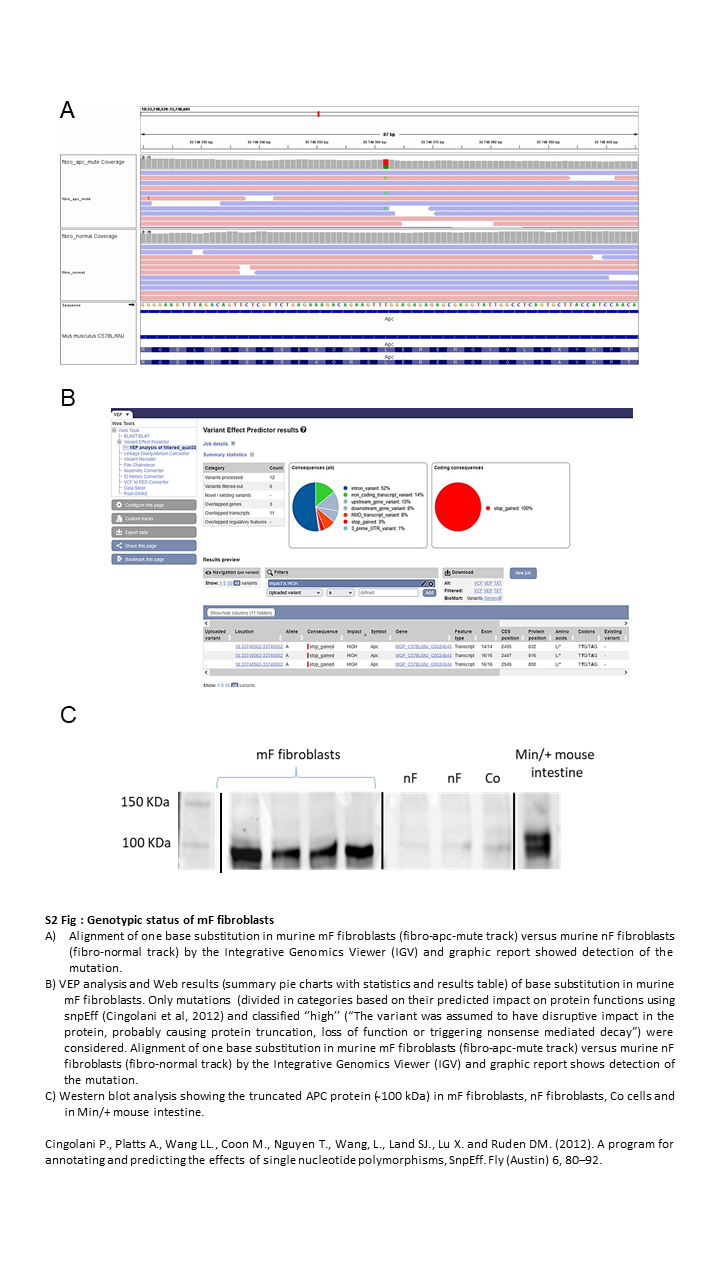

Supplement: S2 Fig — A) Alignment of one base substitution in murine mF fibroblasts (fibro-apc-mute track) versus murine nF fibroblasts (fibro-normal track) by the Integrative Genomics Viewer (IGV) and graphic report showed detection of the mutation. B) VEP analysis and Web results (summary pie charts with statistics and results table) of base substitution in murine mF fibroblasts. Only mutations (divided in categories based on their predicted impact on protein functions using snpEff (Cingolani et al, 2012) and classified ‘‘high” (“The variant was assumed to have disruptive impact in the protein, probably causing protein truncation, loss of function or triggering nonsense mediated decay”) were considered. Alignment of one base substitution in murine mF fibroblasts (fibro-apc-mute track) versus murine nF fibroblasts (fibro-normal track) by the Integrative Genomics Viewer (IGV) and graphic report shows detection of the mutation. C) Western blot analysis showing the truncated APC protein (~100 kDa) in mF fibroblasts, nF fibroblasts, Co cells and in Min/+ mouse intestine. Cingolani P., Platts A., Wang LL., Coon M., Nguyen T., Wang, L., Land SJ., Lu X. and Ruden DM. (2012). A program for annotating and predicting the effects of single nucleotide polymorphisms, SnpEff. Fly (Austin) 6, 80–92. (TIF) [file pone.0273858.s005.tif]

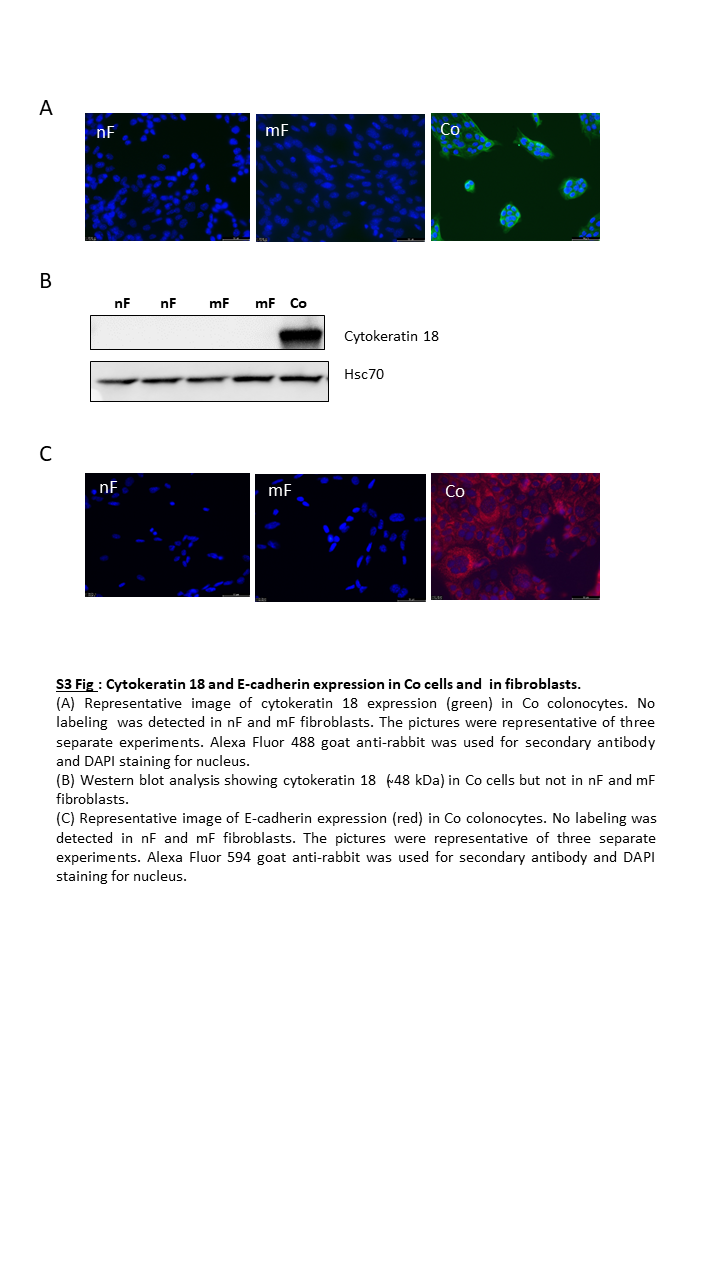

Supplement: S3 Fig — (A) Representative image of cytokeratin 18 expression (green) in Co colonocytes. No labeling was detected in nF and mF fibroblasts. The pictures were representative of three separate experiments. Alexa Fluor 488 goat anti-rabbit was used for secondary antibody and DAPI staining for nucleus. (B) Western blot analysis showing cytokeratin 18 (~48 kDa) in Co cells but not in nF and mF fibroblasts. (C) Representative image of E-cadherin expression (red) in Co colonocytes. No labeling was detected in nF and mF fibroblasts. The pictures were representative of three separate experiments. Alexa Fluor 594 goat anti-rabbit was used for secondary antibody and DAPI staining for nucleus. (TIF) [file pone.0273858.s006.tif]

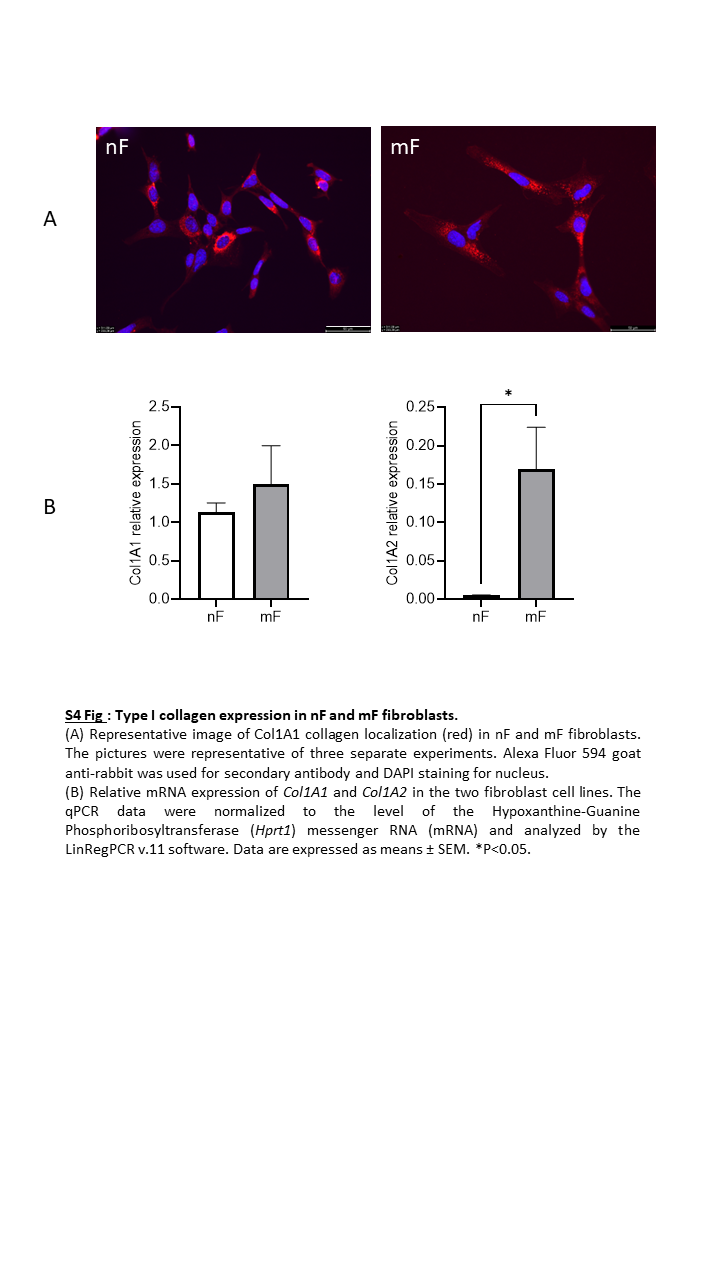

Supplement: S4 Fig — (A) Representative image of Col1A1 collagen localization (red) in nF and mF fibroblasts. The pictures were representative of three separate experiments. Alexa Fluor 594 goat anti-rabbit was used for secondary antibody and DAPI staining for nucleus. (B) Relative mRNA expression of Col1A1 and Col1A2 in the two fibroblast cell lines. The qPCR data were normalized to the level of the Hypoxanthine-Guanine Phosphoribosyltransferase (Hprt1) messenger RNA (mRNA) and analyzed by the LinRegPCR v.11 software. Data are expressed as means ± SEM. *P<0.05. (TIF) [file pone.0273858.s007.tif]

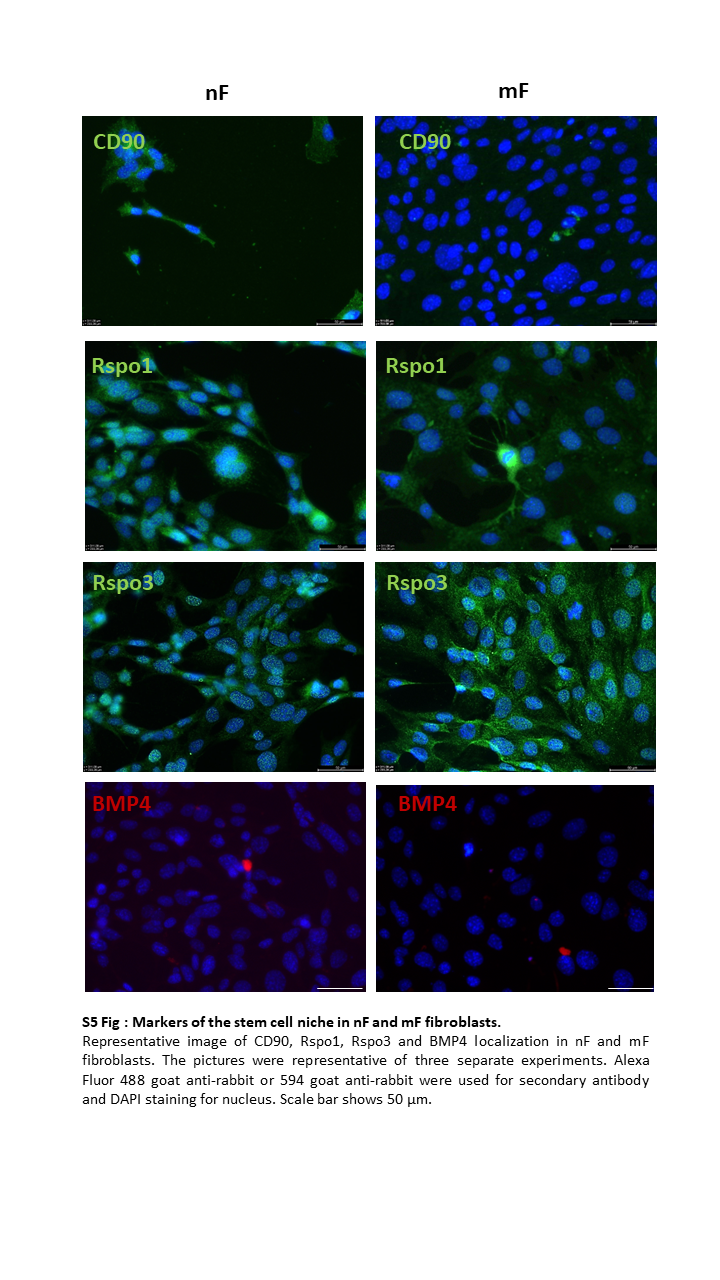

Supplement: S5 Fig — Representative image of CD90, Rspo1, Rspo3 and BMP4 localization in nF and mF fibroblasts. The pictures were representative of three separate experiments. Alexa Fluor 488 goat anti-rabbit or 594 goat anti-rabbit were used for secondary antibody and DAPI staining for nucleus. Scale bar shows 50 μm. (TIF) [file pone.0273858.s008.tif]

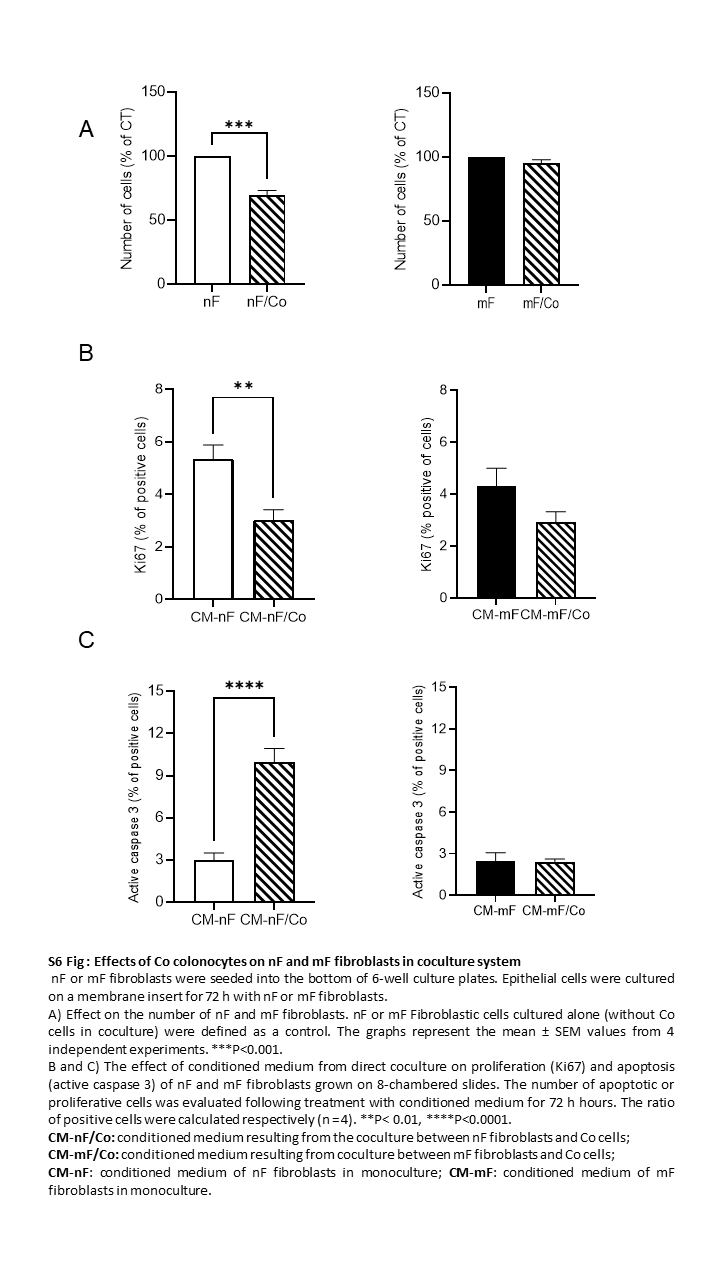

Supplement: S6 Fig — nF or mF fibroblasts were seeded into the bottom of 6-well culture plates. Epithelial cells were cultured on a membrane insert for 72 h with nF or mF fibroblasts. A) Effect on the number of nF and mF fibroblasts. nF or mF Fibroblastic cells cultured alone (without Co cells in coculture) were defined as a control. The graphs represent the mean ± SEM values from 4 independent experiments. ***P<0.001. B and C) The effect of conditioned medium from direct coculture on proliferation (Ki67) and apoptosis (active caspase 3) of nF and mF fibroblasts grown on 8-chambered slides. The number of apoptotic or proliferative cells was evaluated following treatment with conditioned medium for 72 h hours. The ratio of positive cells were calculated respectively (n = 4). **P< 0.01, ****P<0.0001. CM-nF/Co: conditioned medium resulting from the coculture between nF fibroblasts and Co cells; CM-mF/Co: conditioned medium resulting from coculture between mF fibroblasts and Co cells; CM-nF: conditioned medium of nF fibroblasts in monoculture; CM-mF: conditioned medium of mF fibroblasts in monoculture. (TIF) [file pone.0273858.s009.tif]

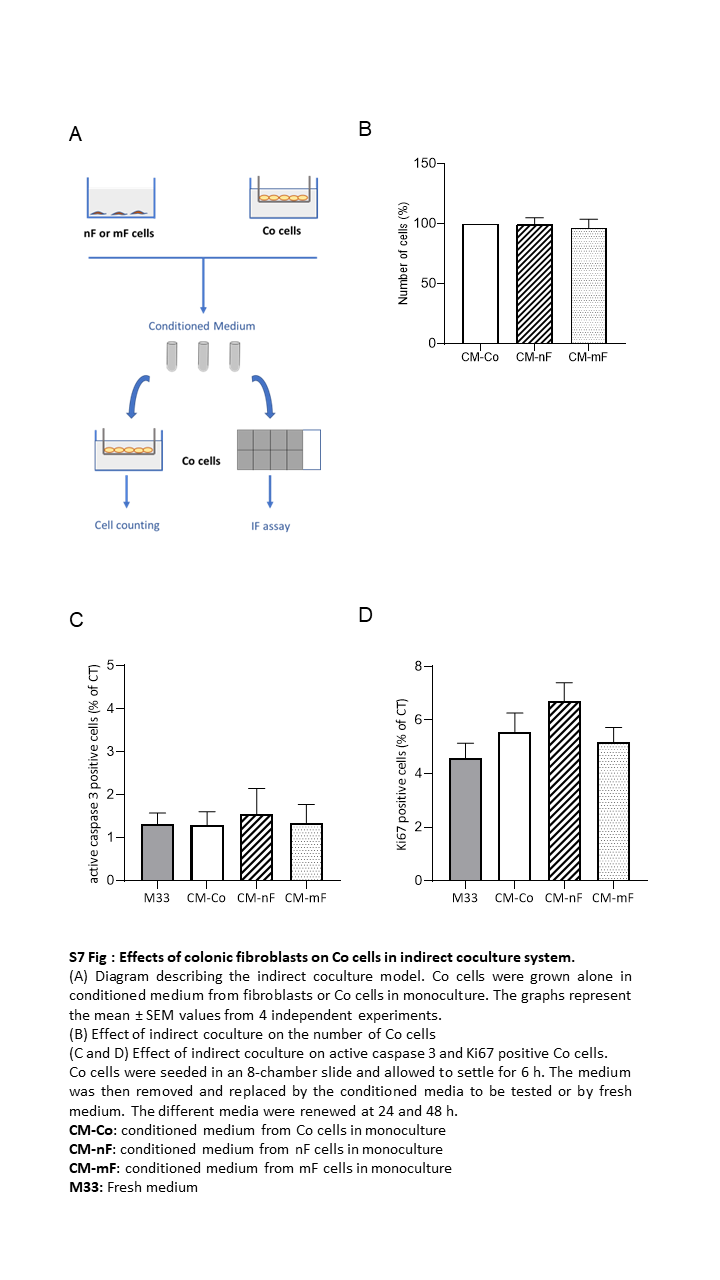

Supplement: S7 Fig — (A) Diagram describing the indirect coculture model. Co cells were grown alone in conditioned medium from fibroblasts or Co cells in monoculture. The graphs represent the mean ± SEM values from 4 independent experiments. (B) Effect of indirect coculture on the number of Co cells. (C and D) Effect of indirect coculture on active caspase 3 and Ki67 positive Co cells. Co cells were seeded in an 8-chamber slide and allowed to settle for 6 h. The medium was then removed and replaced by the conditioned media to be tested or by fresh medium. The different media were renewed at 24 and 48 h. CM-Co: conditioned medium from Co cells in monoculture. CM-nF: conditioned medium from nF cells in monoculture. CM-mF: conditioned medium from mF cells in monoculture. M33: Fresh medium. (TIF) [file pone.0273858.s010.tif]

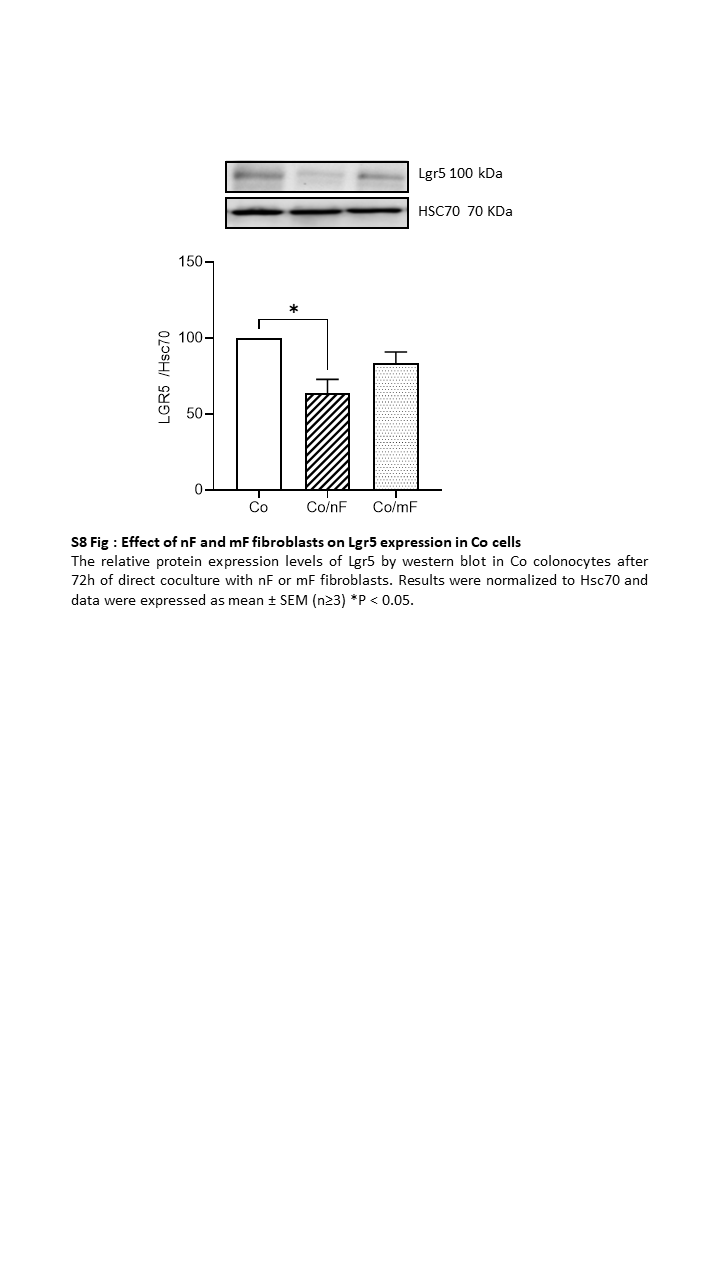

Supplement: S8 Fig — The relative protein expression levels of Lgr5 by western blot in Co colonocytes after 72h of direct coculture with nF or mF fibroblasts. Results were normalized to Hsc70 and data were expressed as mean ± SEM (n≥3) *P < 0.05. (TIF) [file pone.0273858.s011.tif]

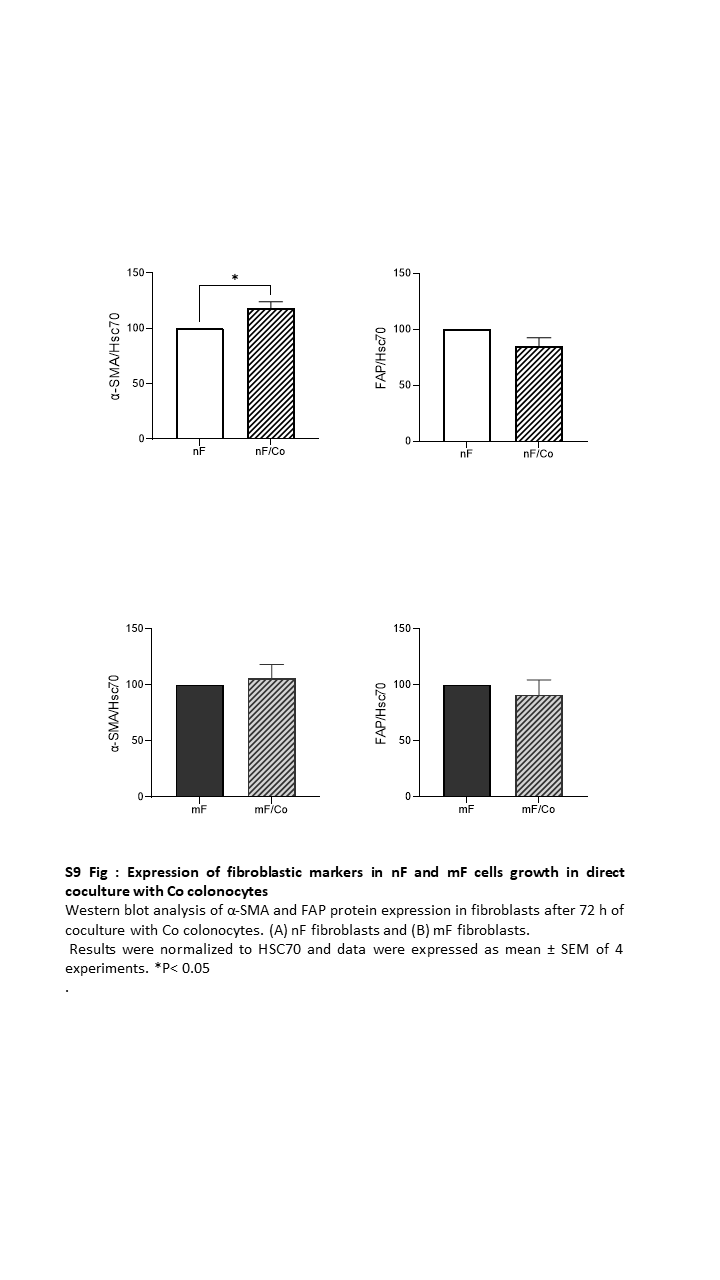

Supplement: S9 Fig — Western blot analysis of α-SMA and FAP protein expression in fibroblasts after 72 h of coculture with Co colonocytes. (A) nF fibroblasts and (B) mF fibroblasts. Results were normalized to HSC70 and data were expressed as mean ± SEM of 4 experiments. *P< 0.05. (TIF) [file pone.0273858.s012.tif]
