# Supplementary material for: Study of the colonic epithelial-mesenchymal dialogue through establishment of two activated or not mesenchymal cell lines: Activated and resting ones differentially modulate colonocytes in co-culture
Source: PLoS One. 2022 Aug 30;17(8):e0273858. doi: 10.1371/journal.pone.0273858 (PMC9426876; doi:10.1371/journal.pone.0273858)

Characterization of nF and mF cell lines

α-SMA (42 kDa)

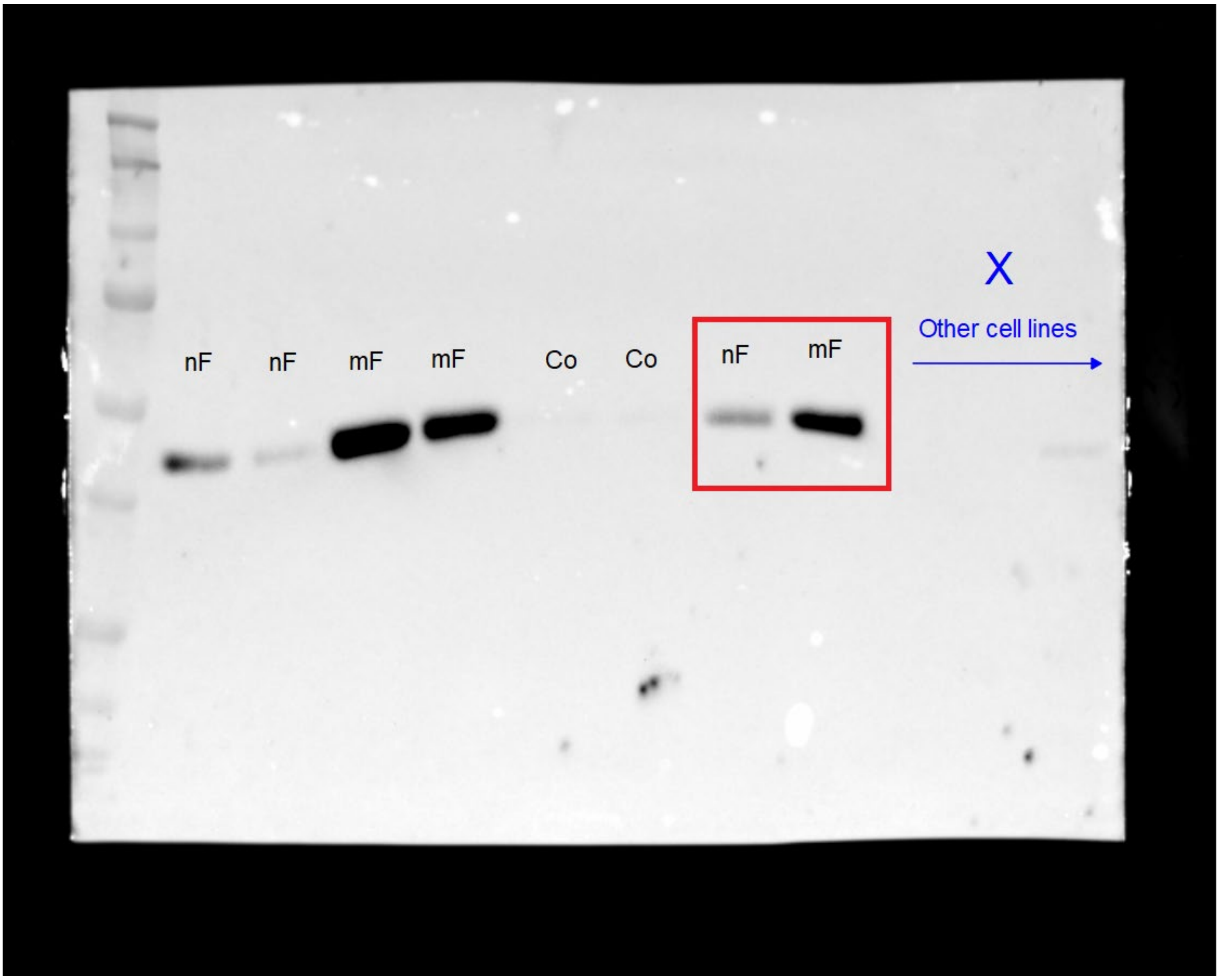

HSC70 for αSMA, vimentin and cytokeratin 18

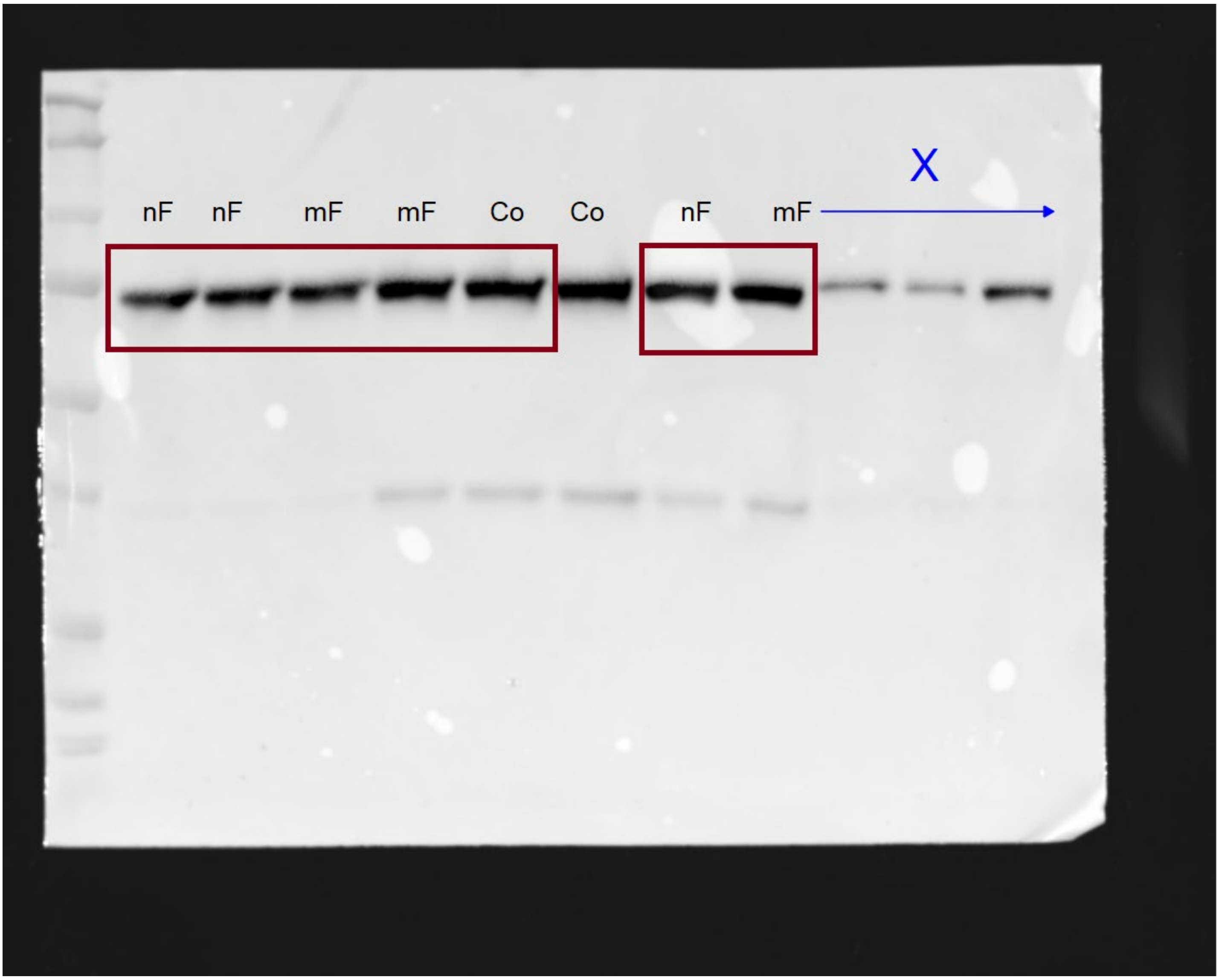

Cytokeratin 18 (45 kDa)

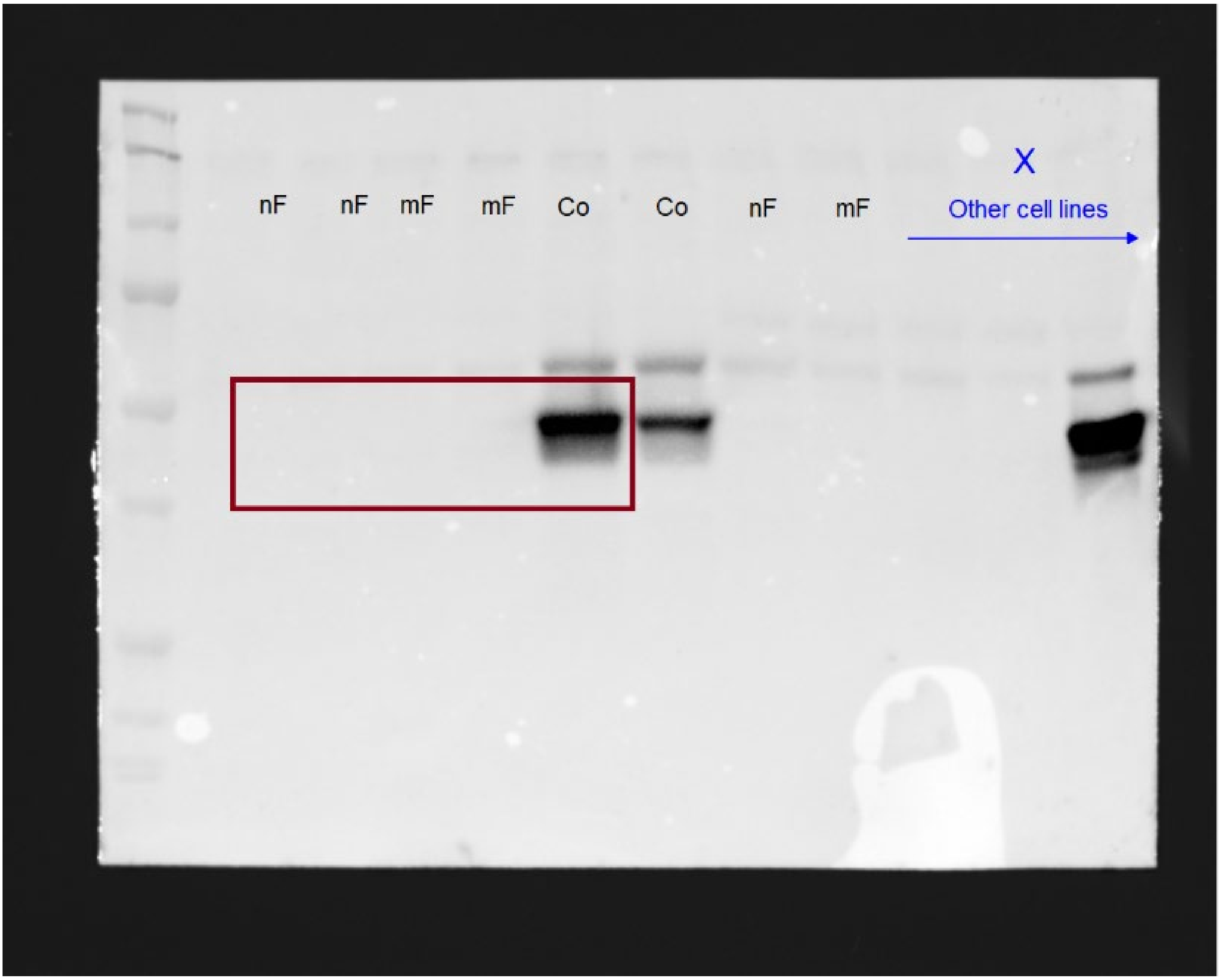

Vimentin (54 kDa)

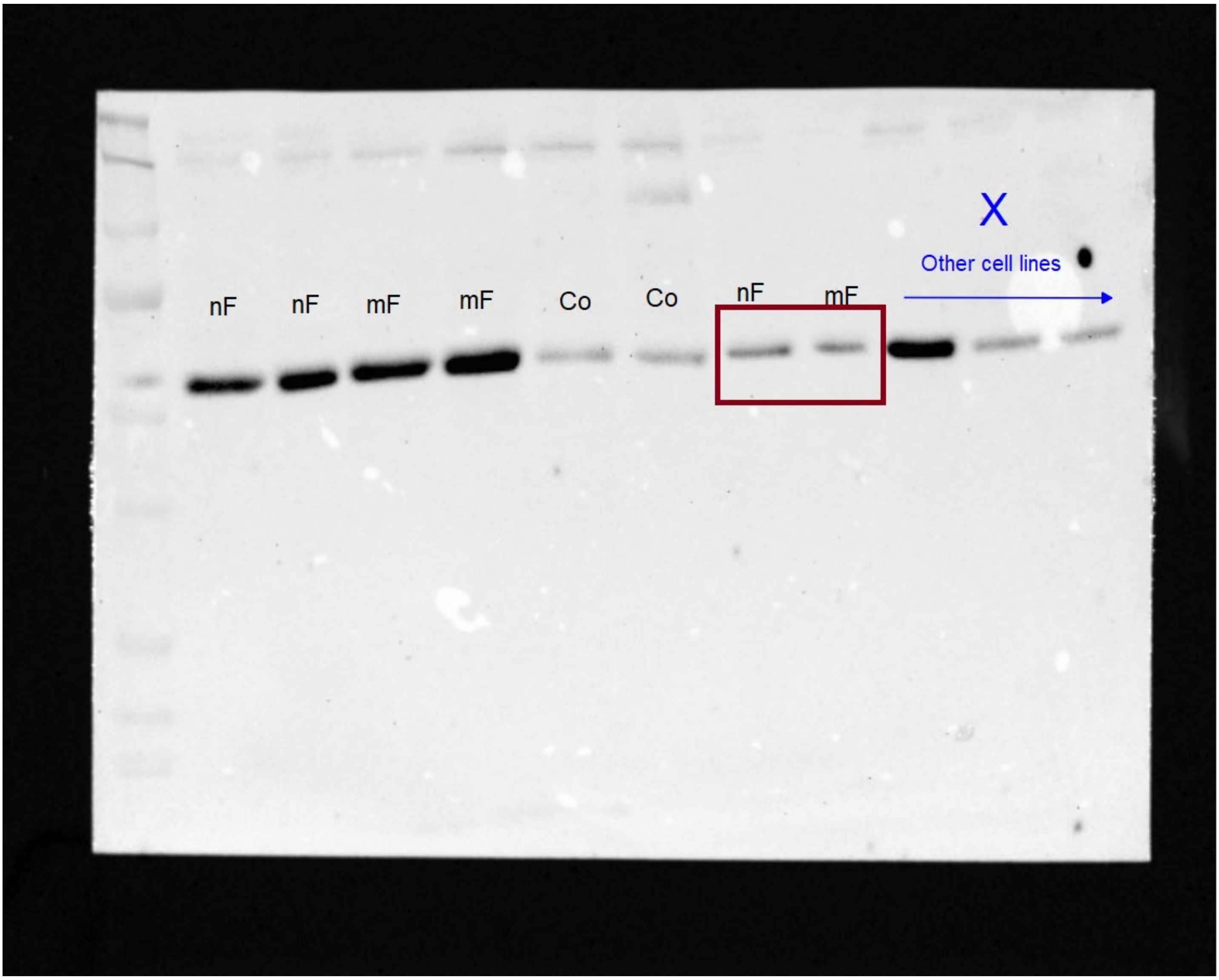

FAP (approx. 95 kDa)

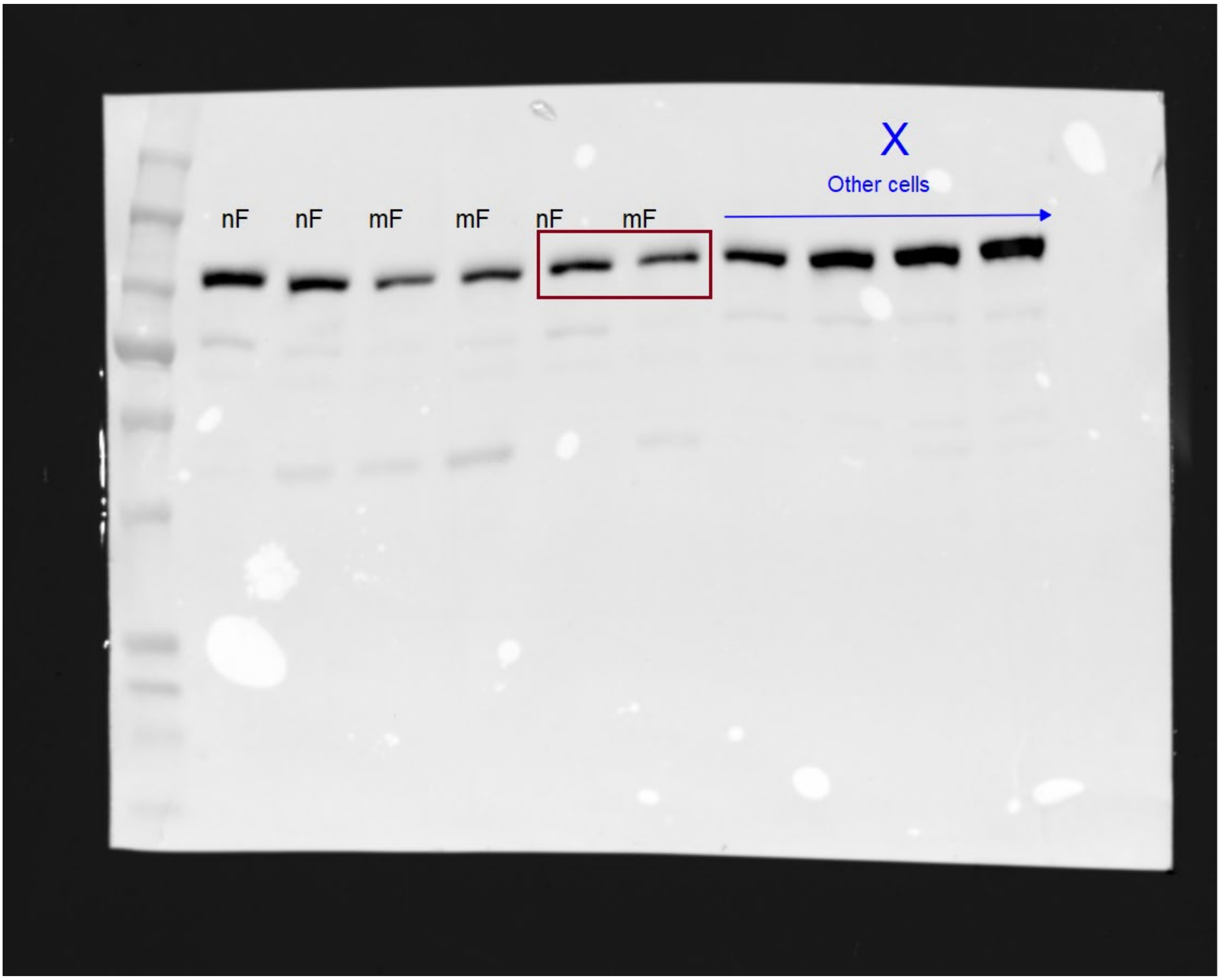

HSC70 for FAP

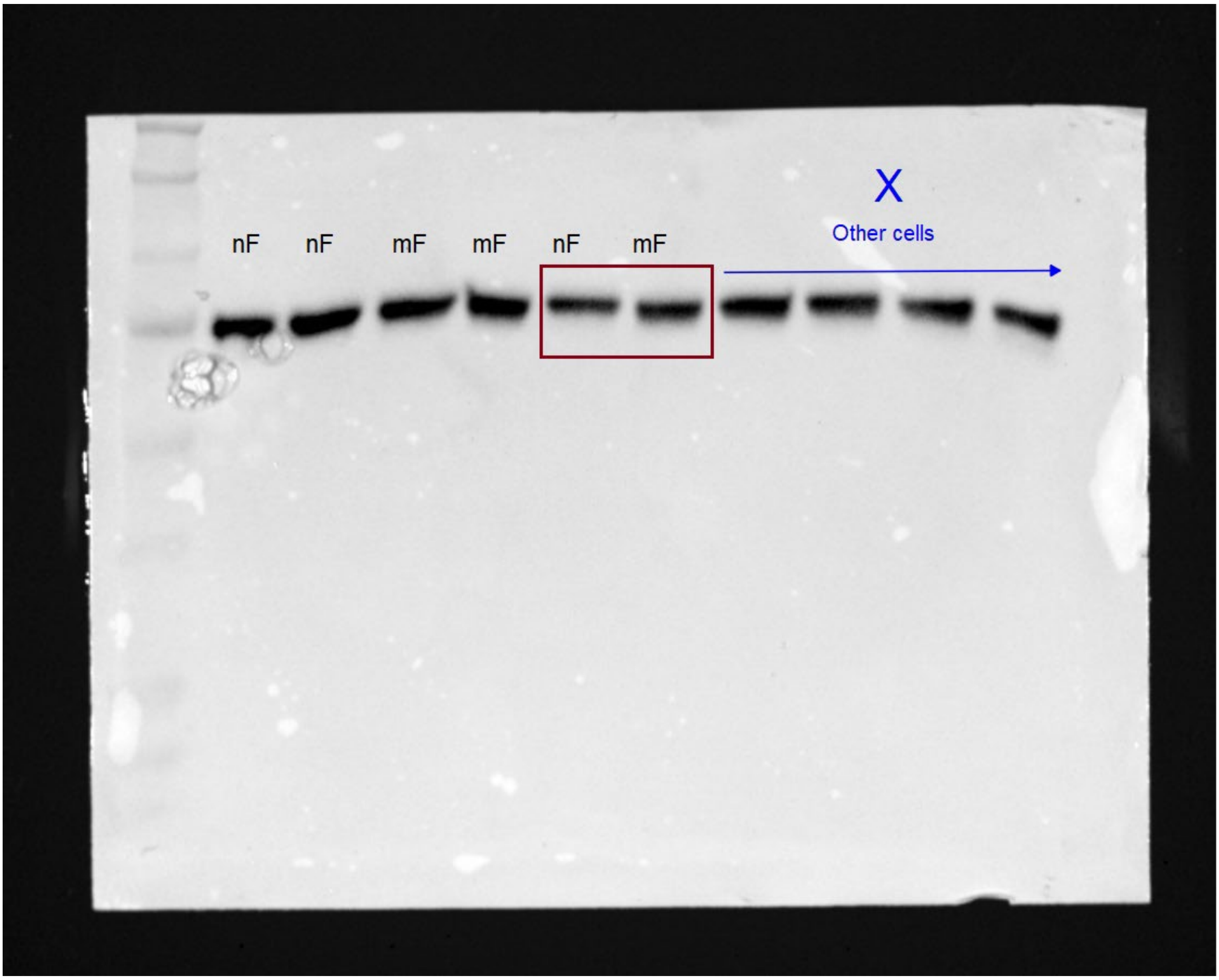

Effect of nF and mF fibroblasts on the phenotype of colonocytes

Cytokeratin 18 (45 kDa)

Blot 1

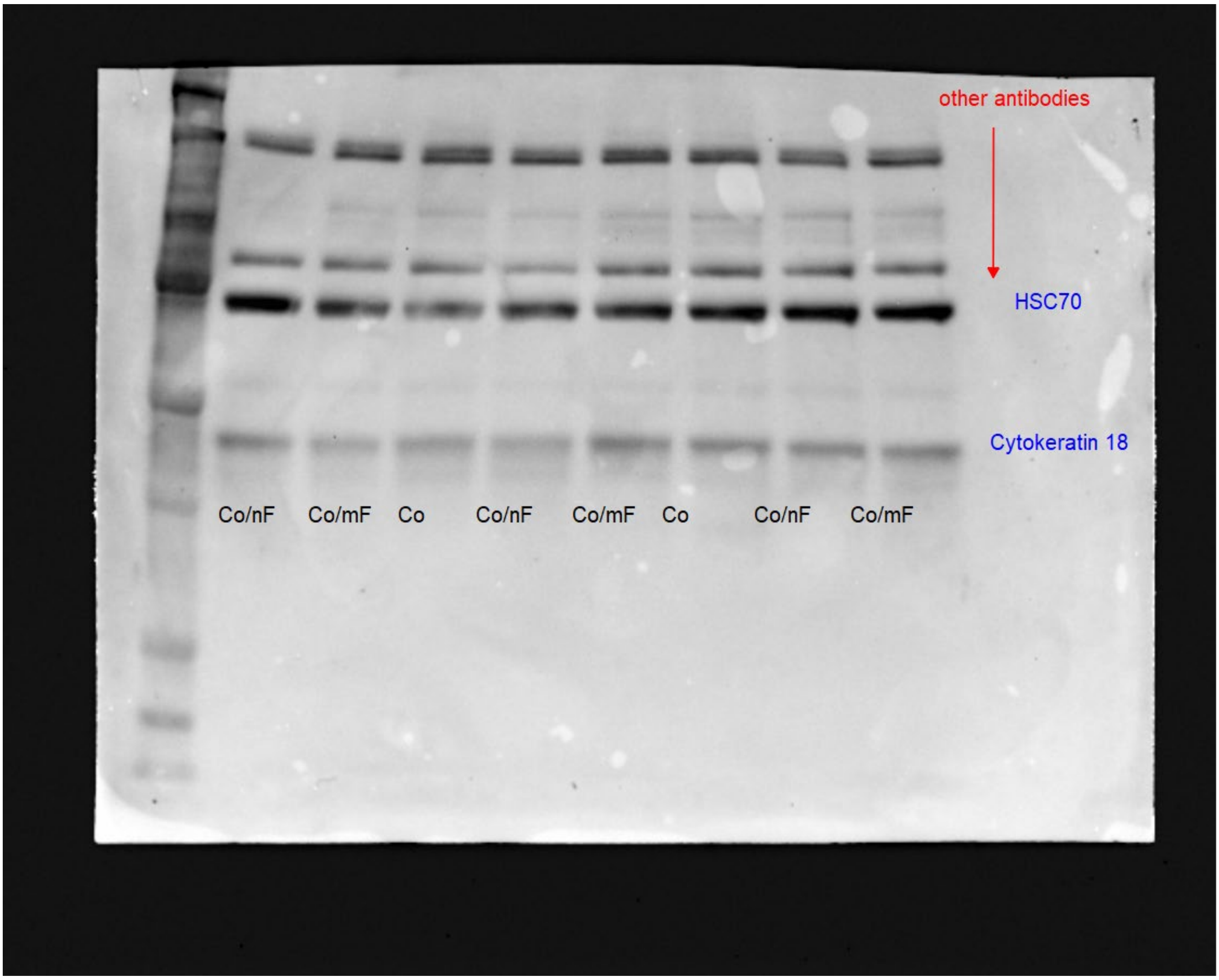

Cytokeratin 18: Blot 2

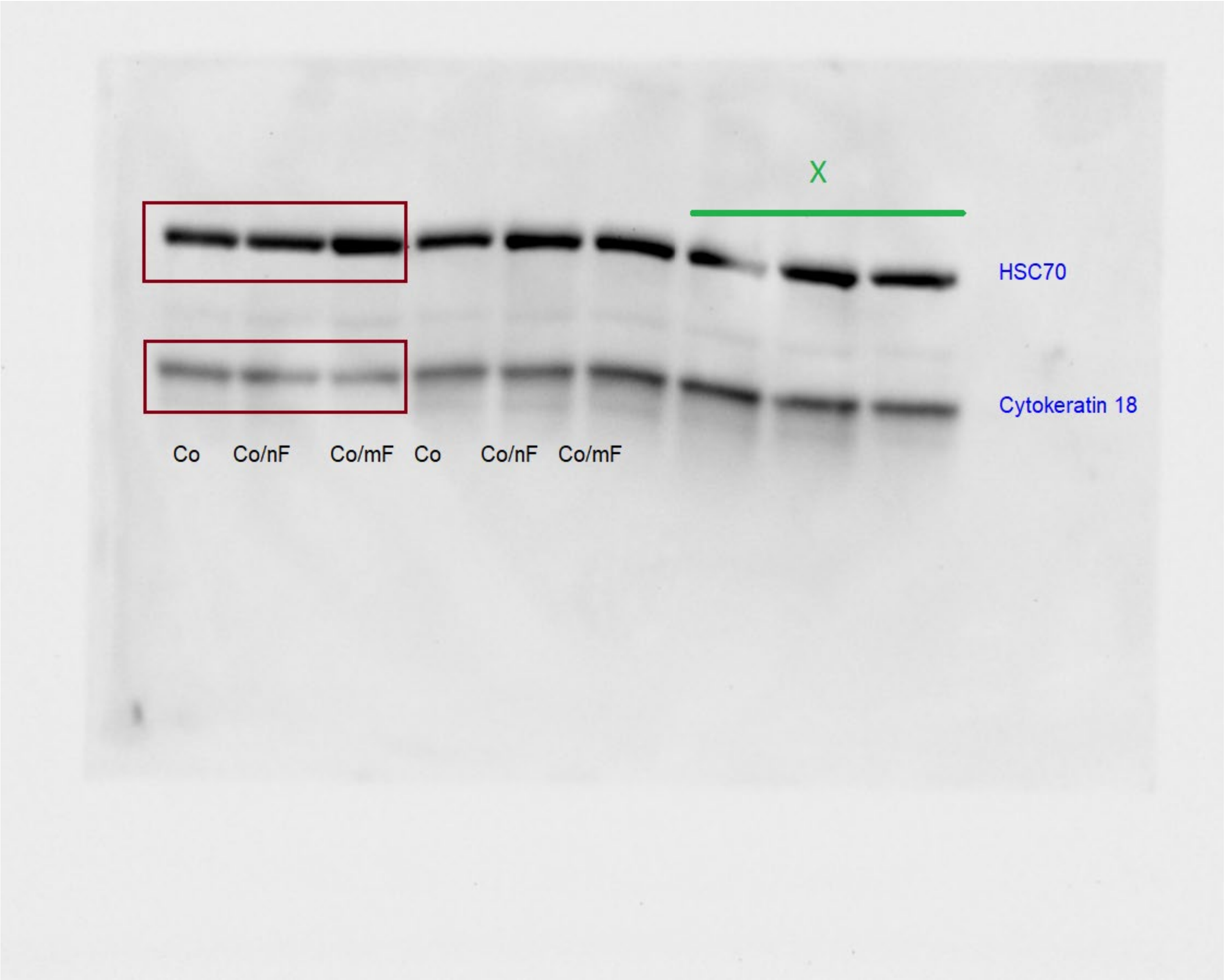

E-cadherin (approx. 120 kDa)

Blot 1

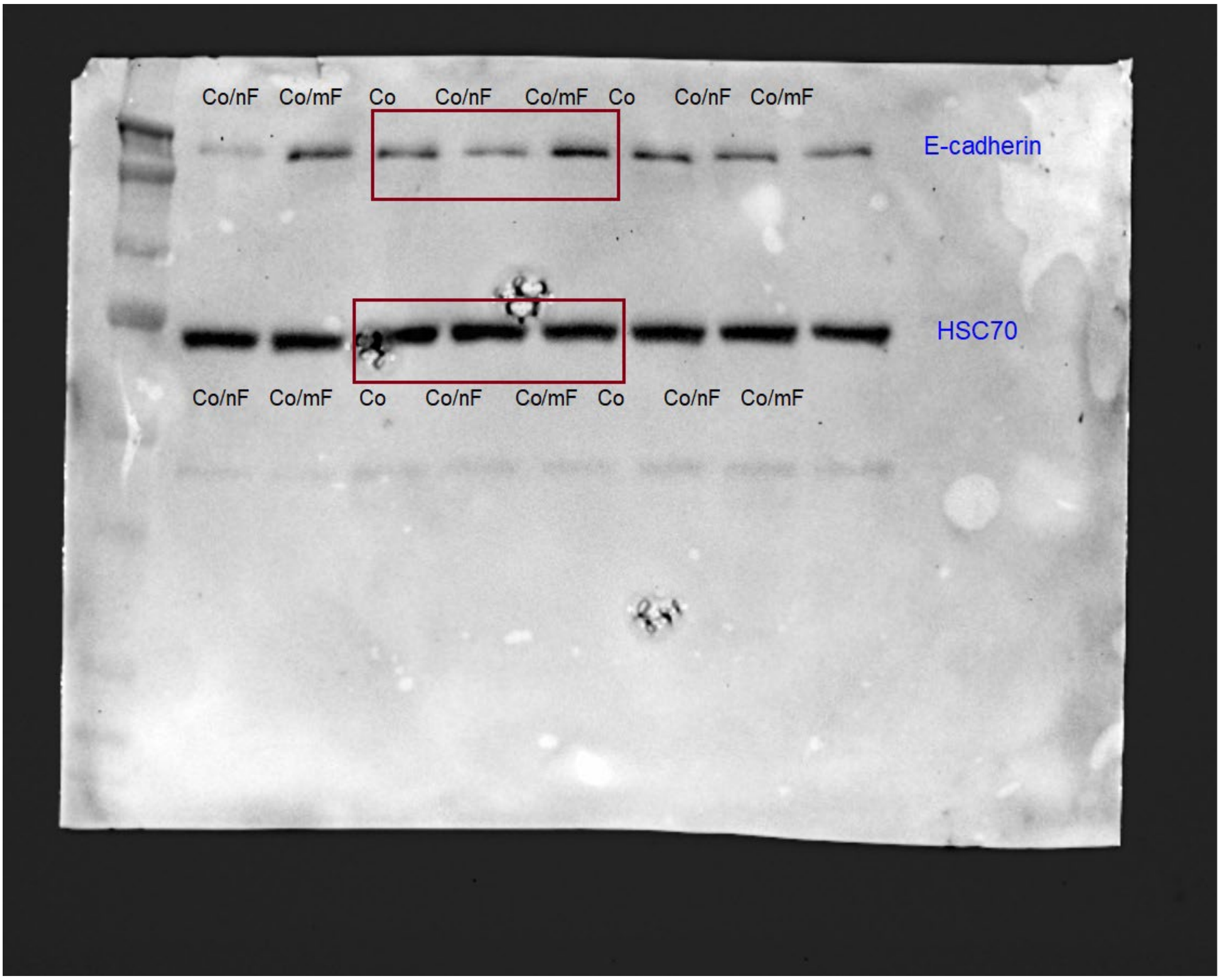

Blot 2 E-cadherin

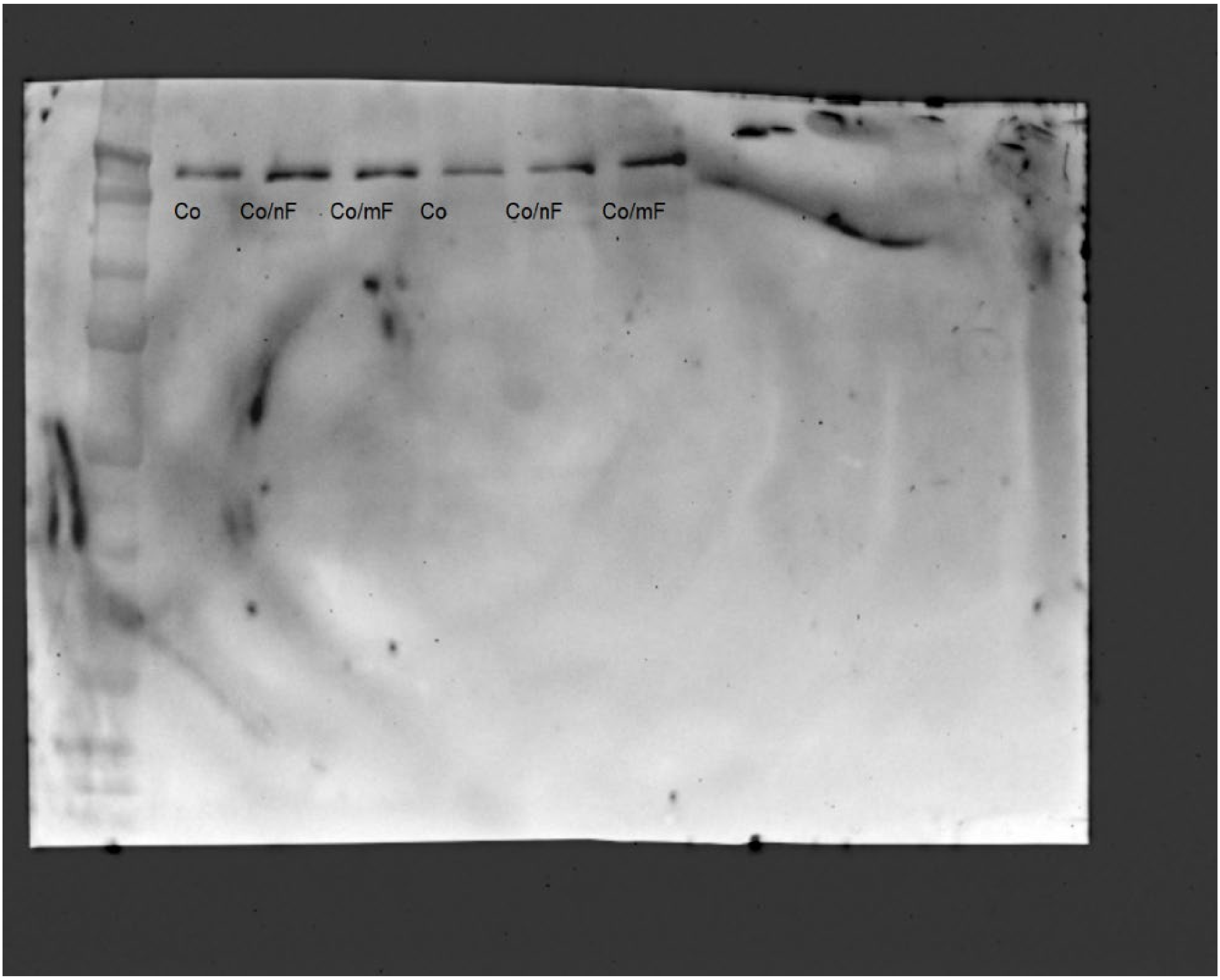

HSC70 for blot 2

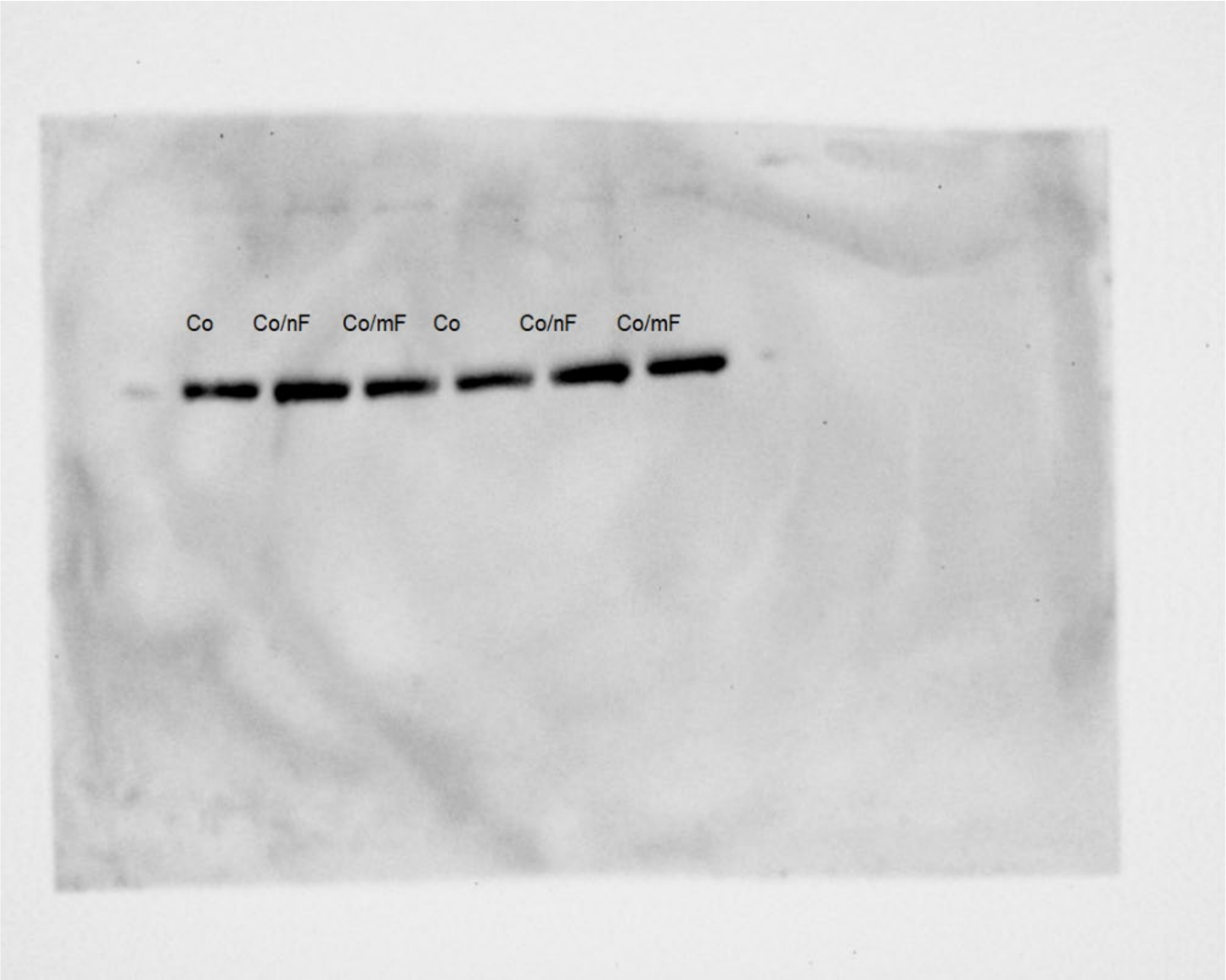

LGR5 100 kDa

Blot 1

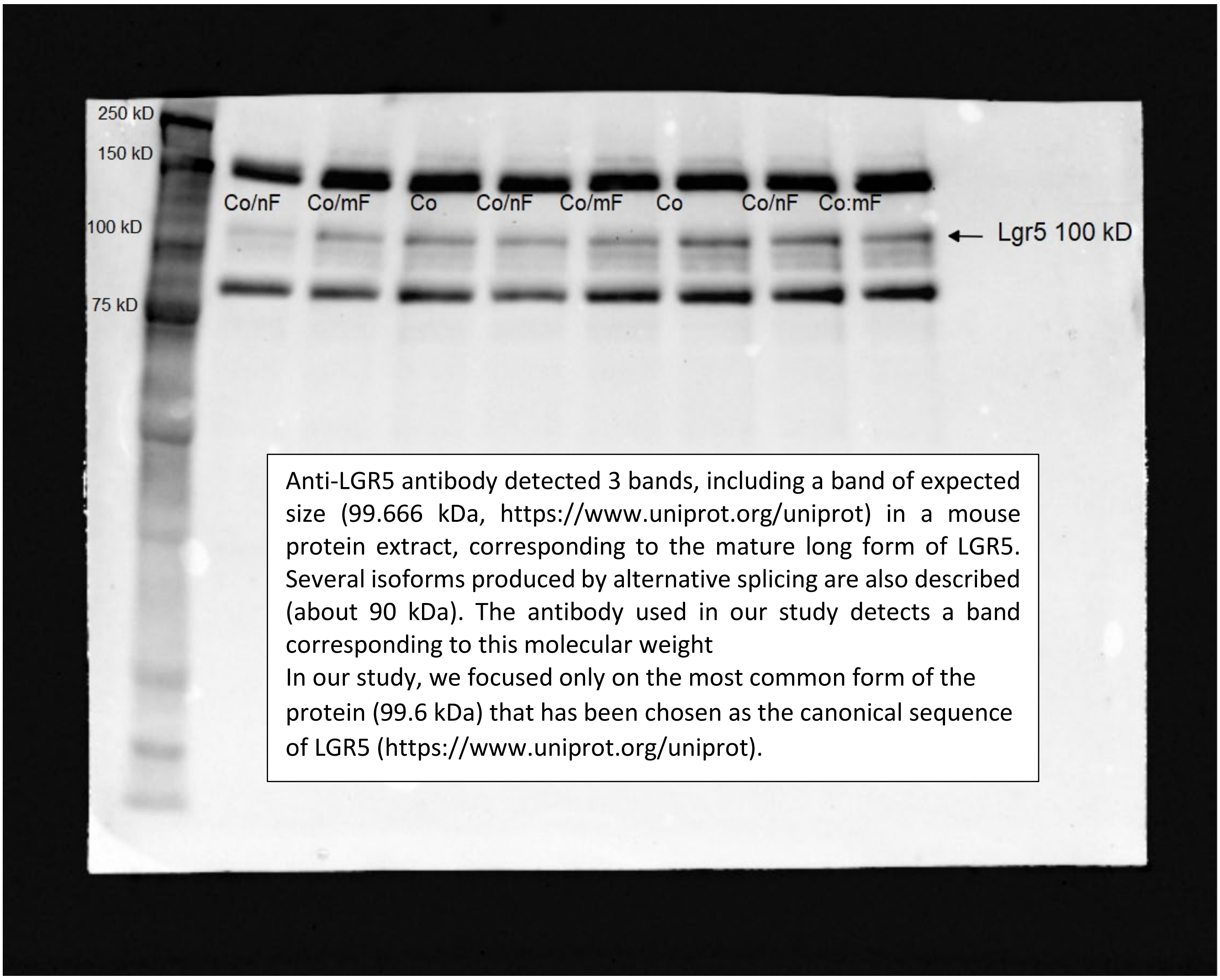

HSC70 for Lgr5

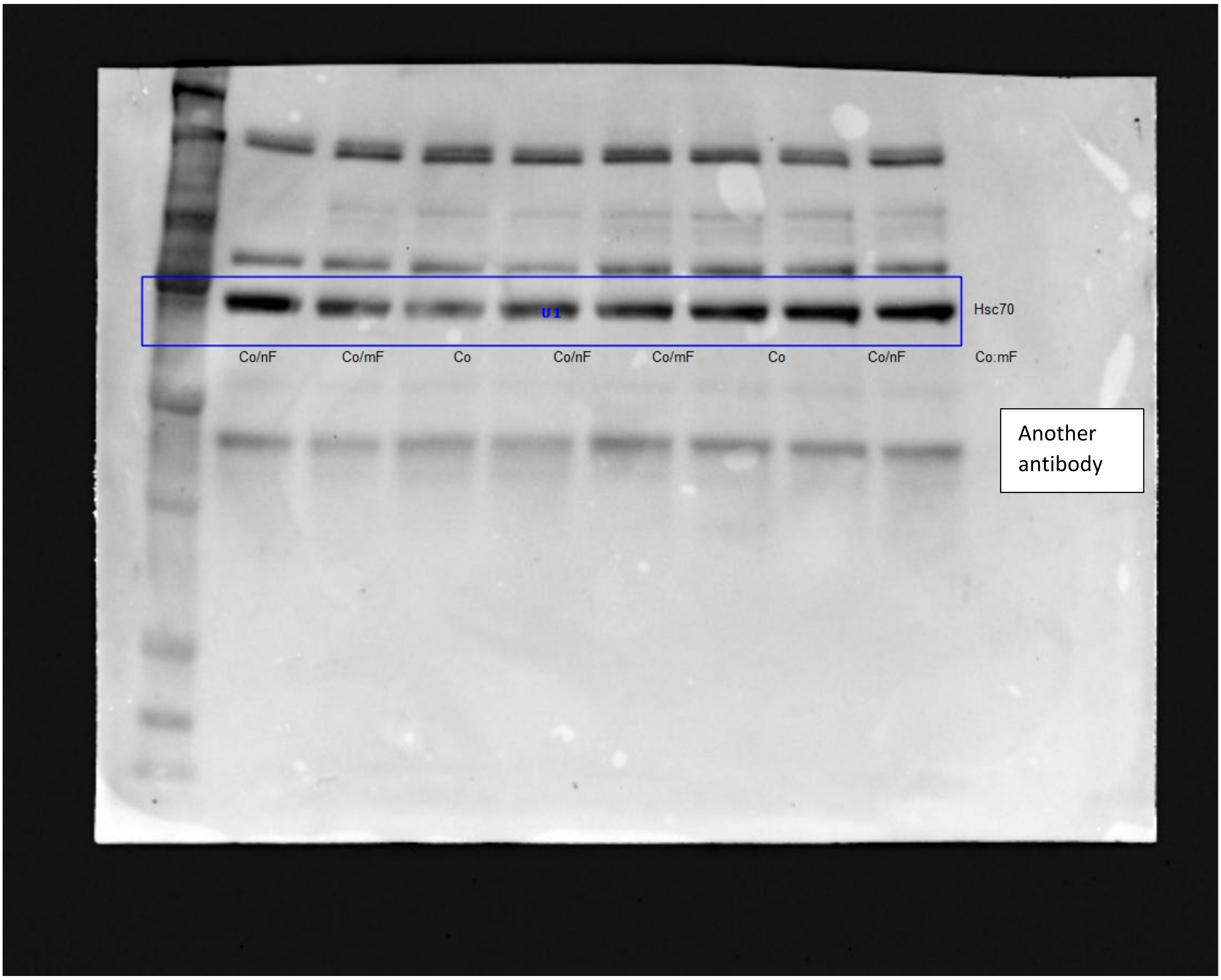

Expression of fibroblastic markers in nF and mF cells growth in direct coculture with Co colonocytes

α-SMA

<u>**αSMA: Blot_1**</u>

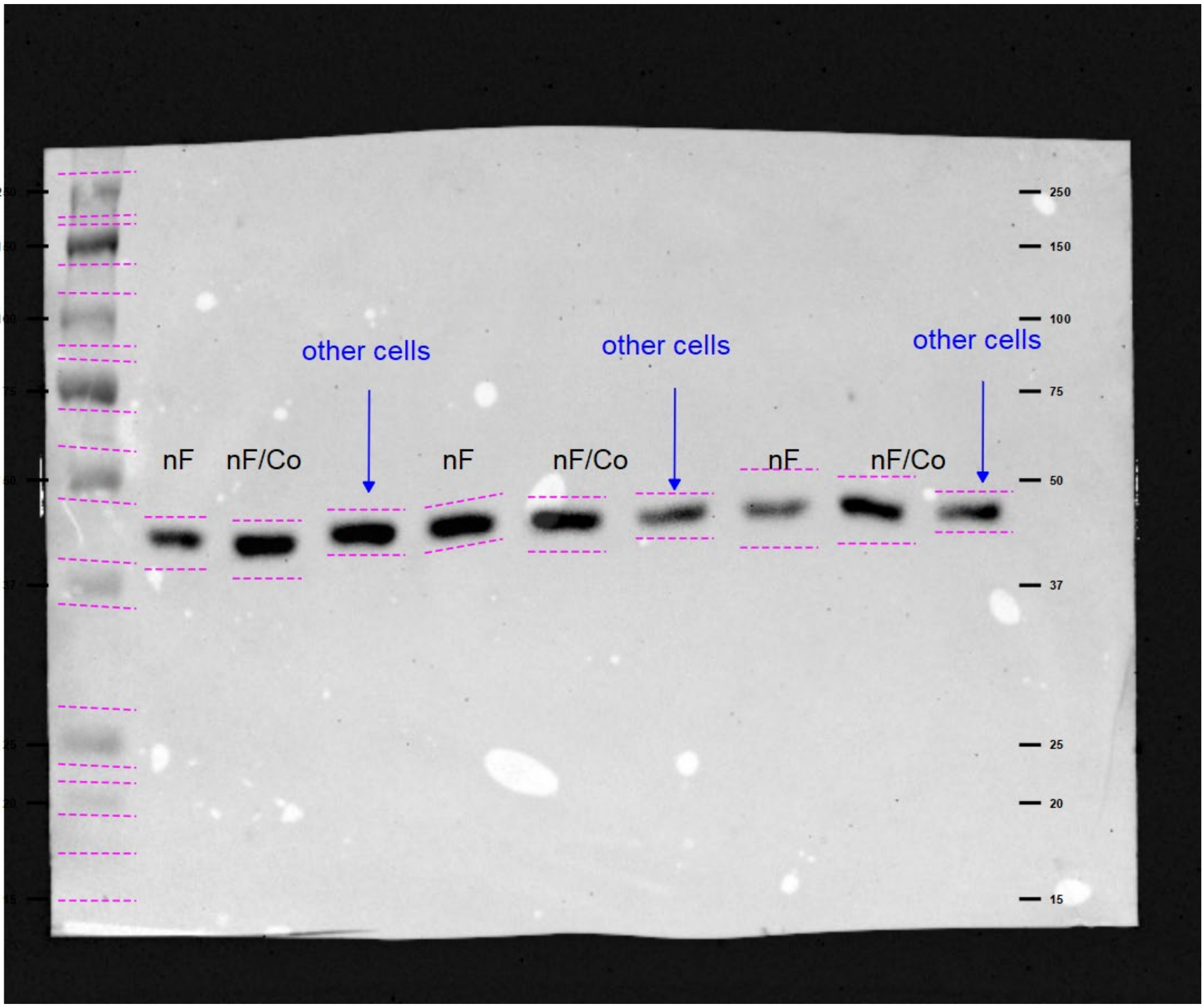

αSMA: Blot 2

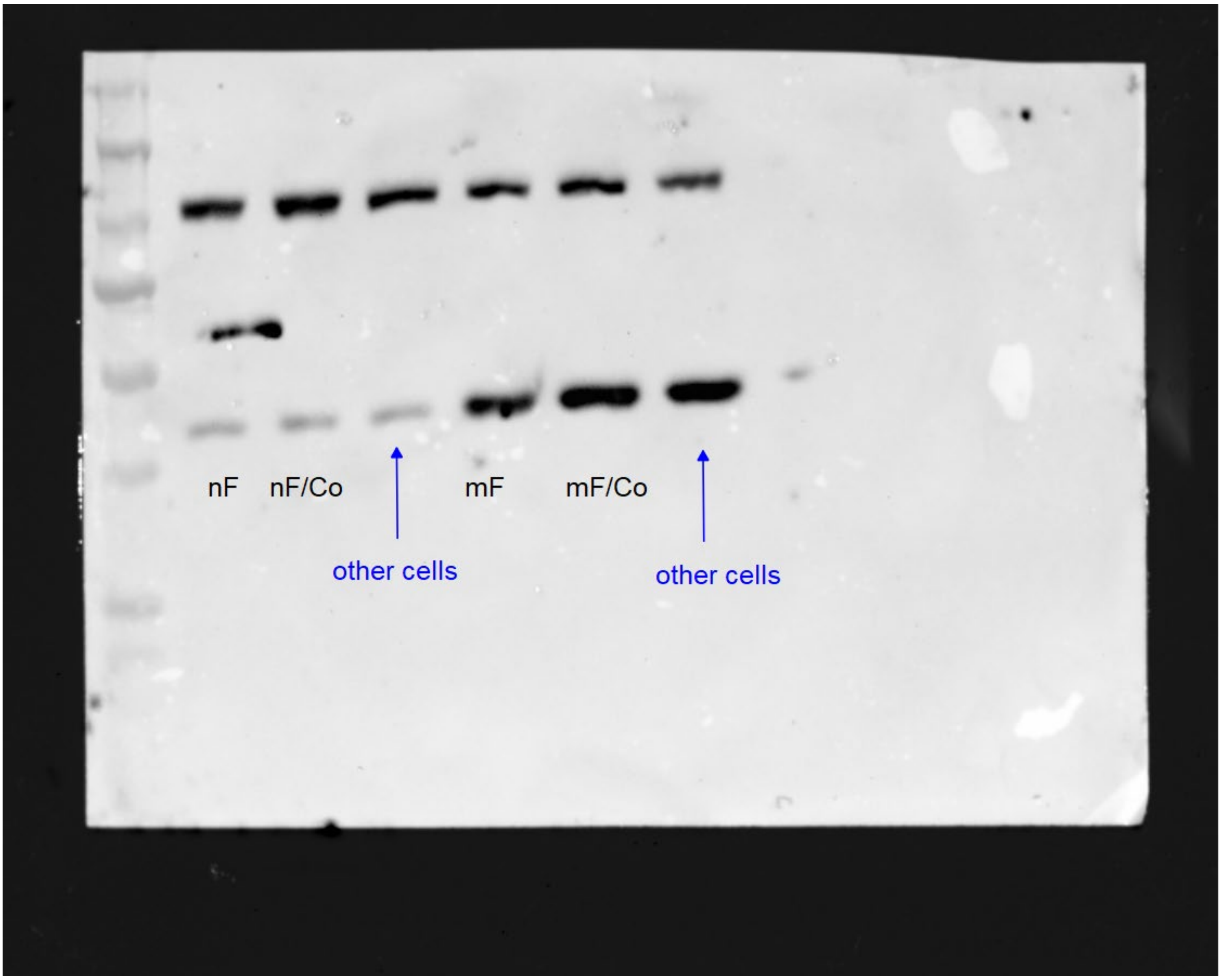

αSMA : Blot 3

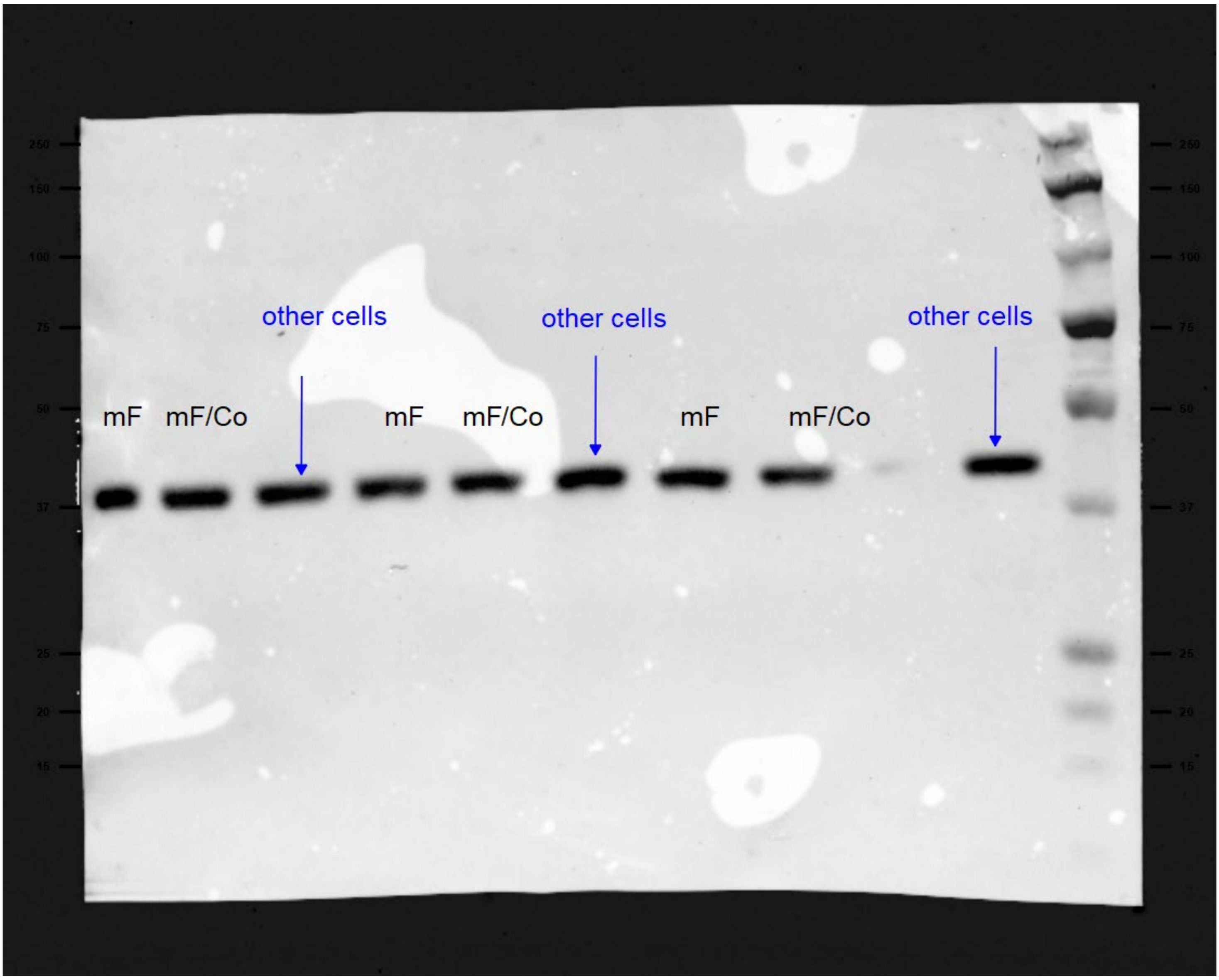
250
150
100
75
50
37
25
20
15
other cells
other cells
other cells
mF
mF/Co
mF
mF/Co
mF
mF/Co

HSC70 for blot 1 αSMA and blot 1 FAP

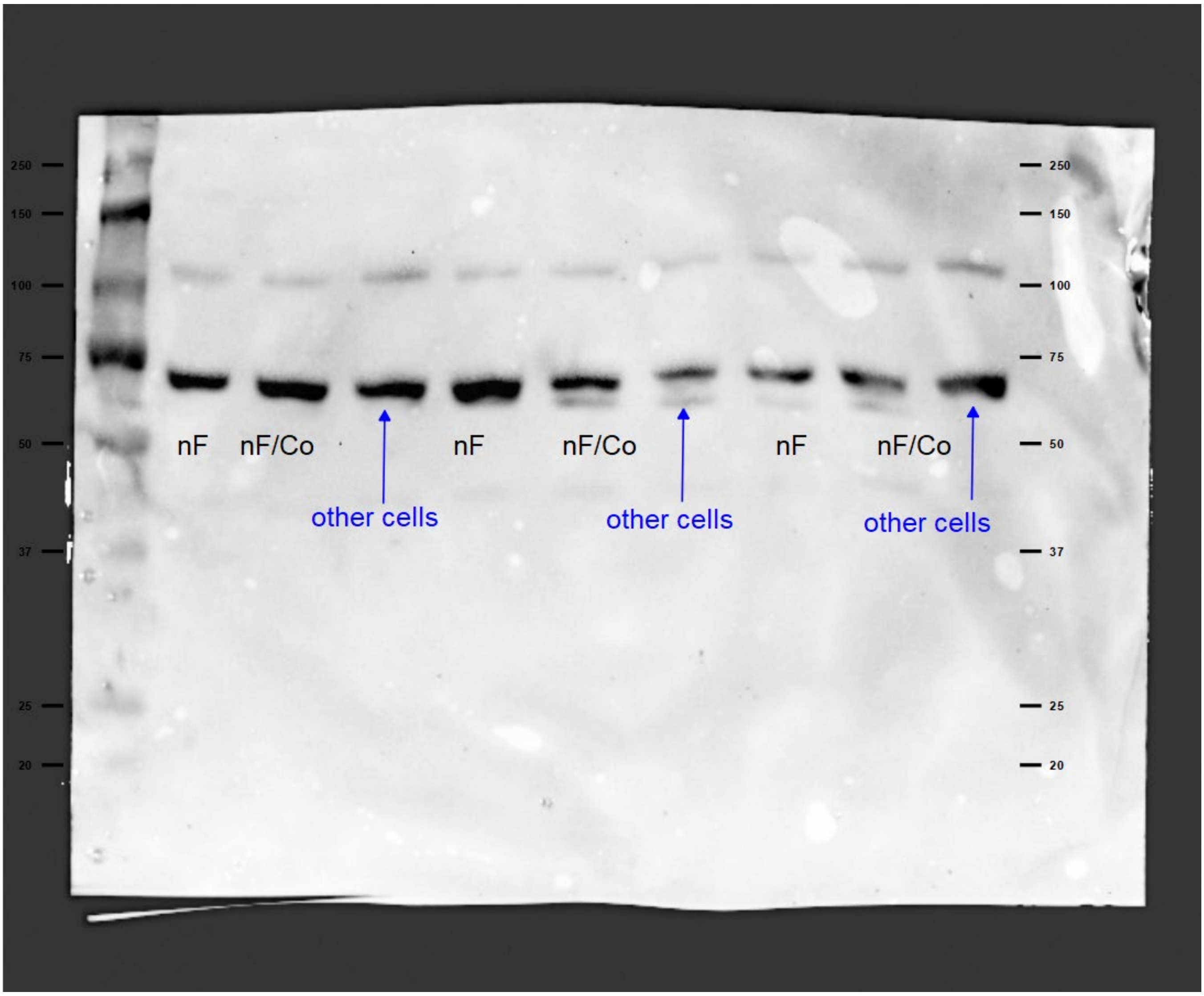

HSC70 for gel 2 αSMA and gel 2 FAP

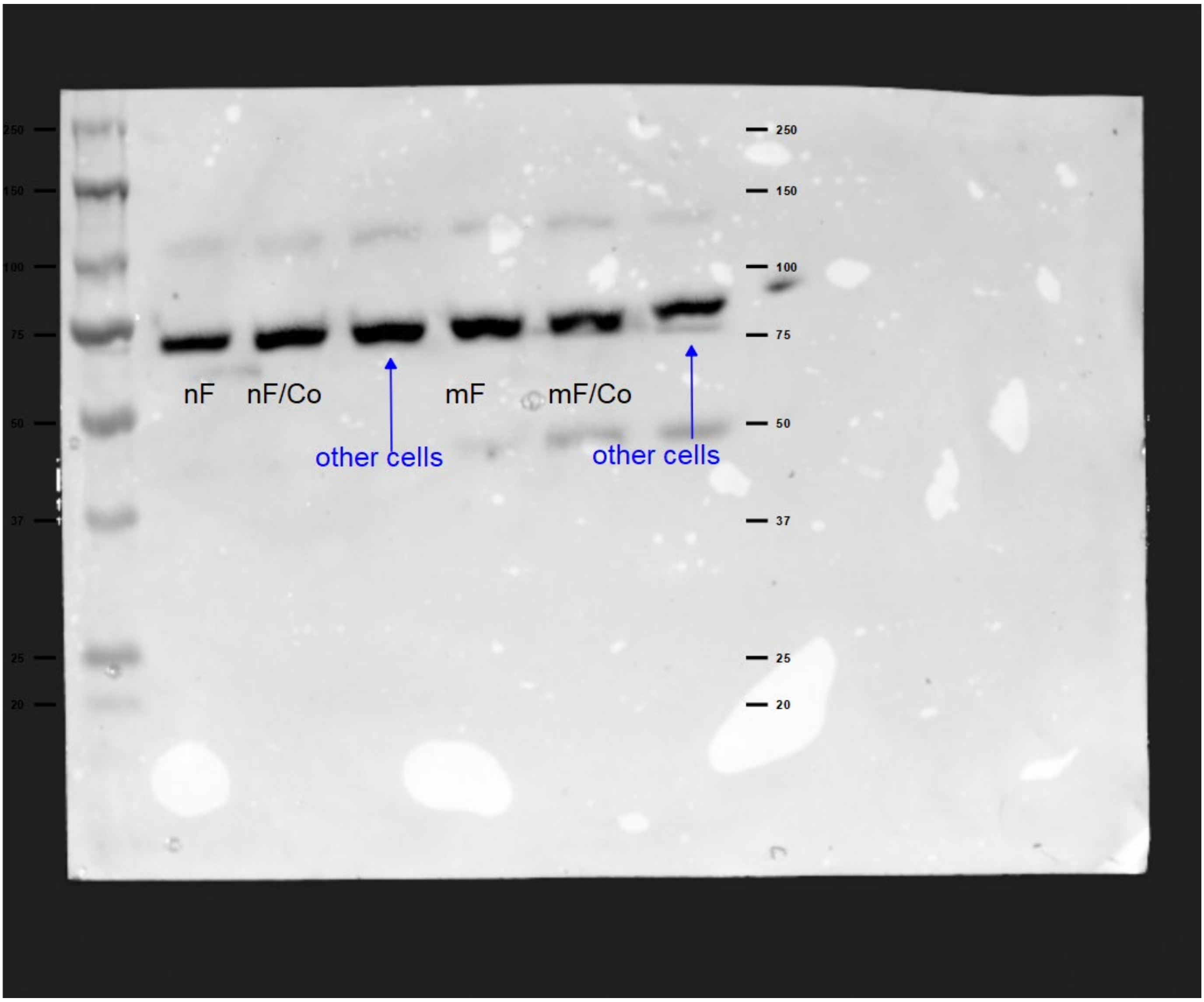

HSC70 for blot 3 αSMA and blot 3 FAP

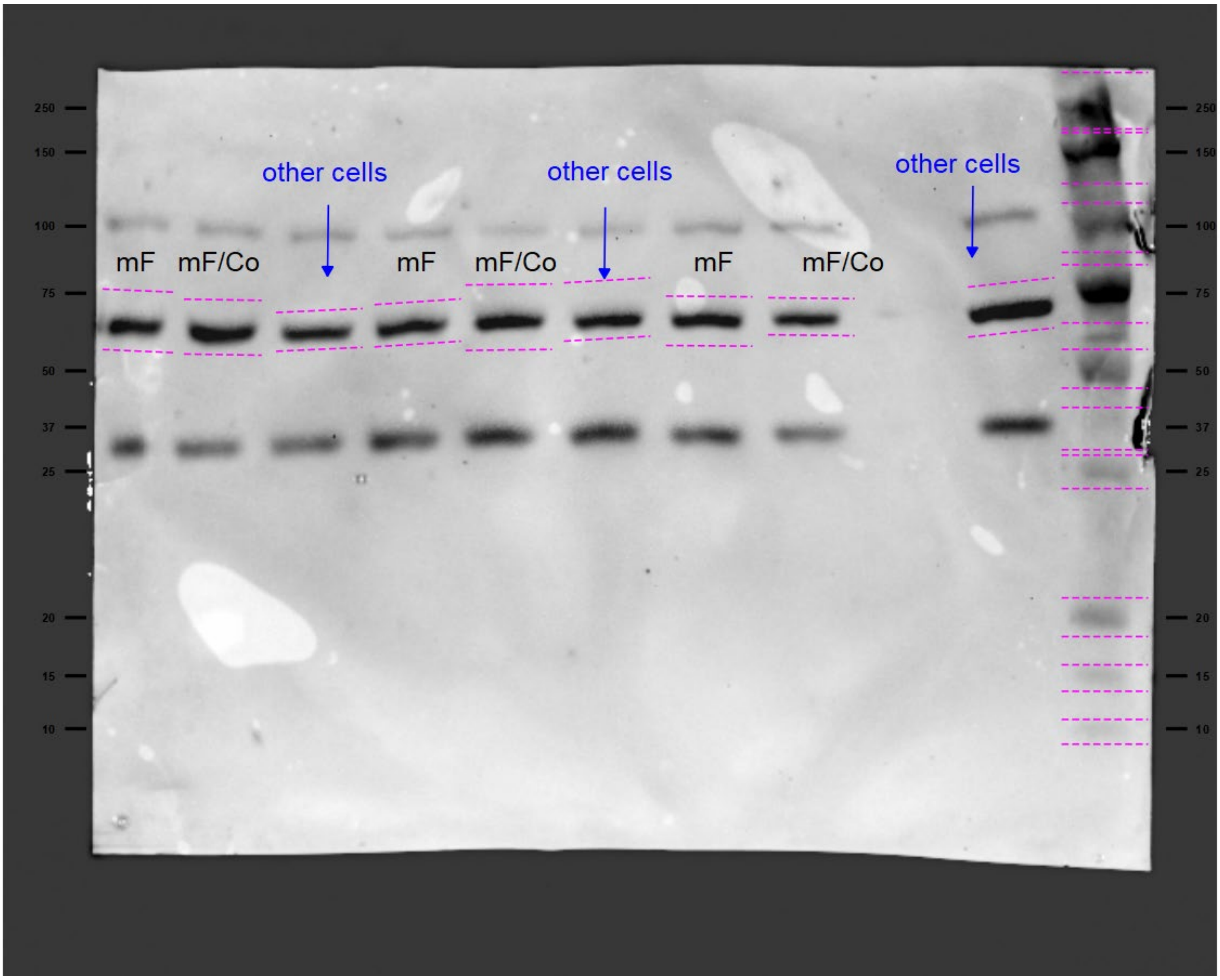

FAP

<u>Blot 1 FAP</u>

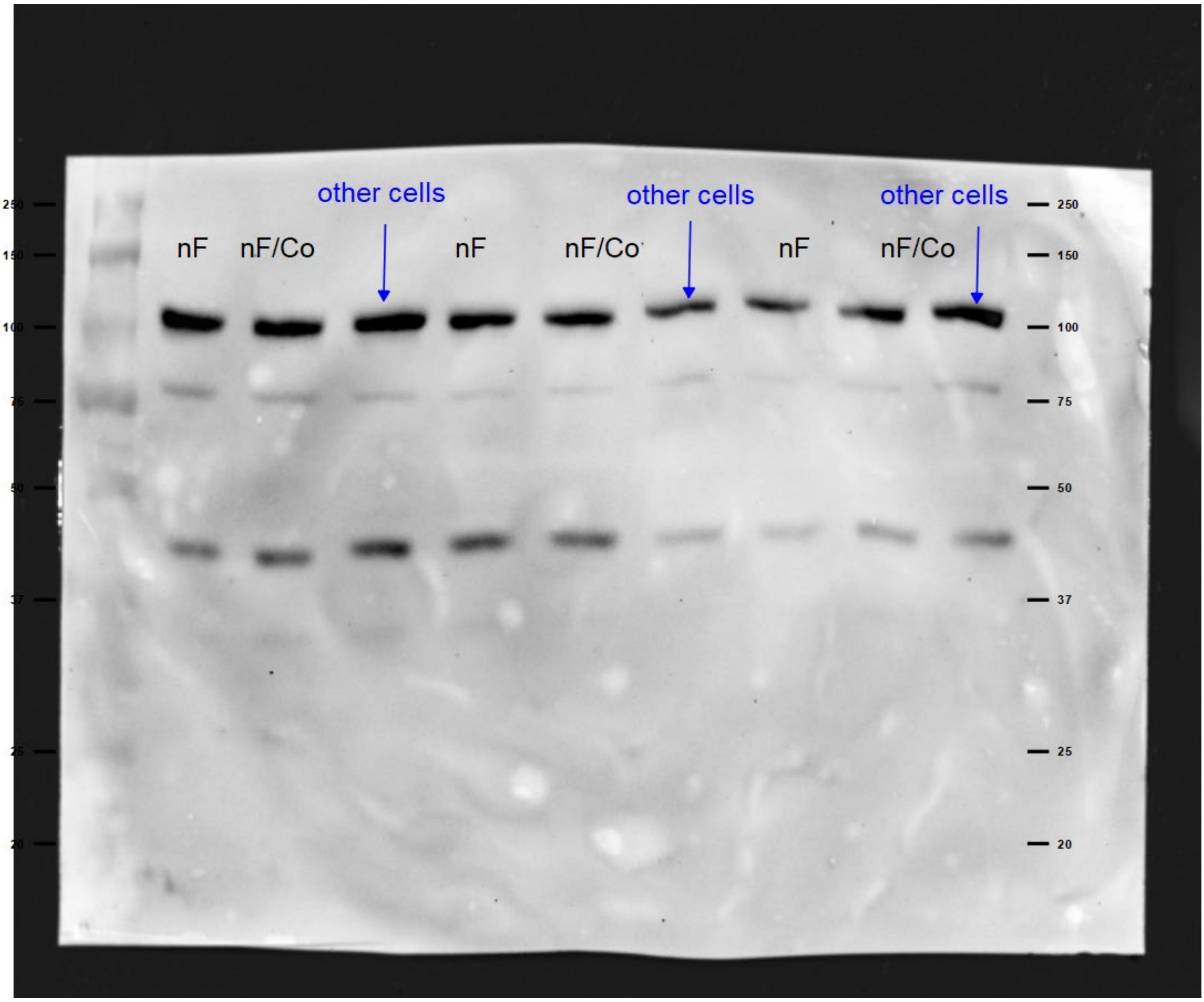

Blot 2 FAP

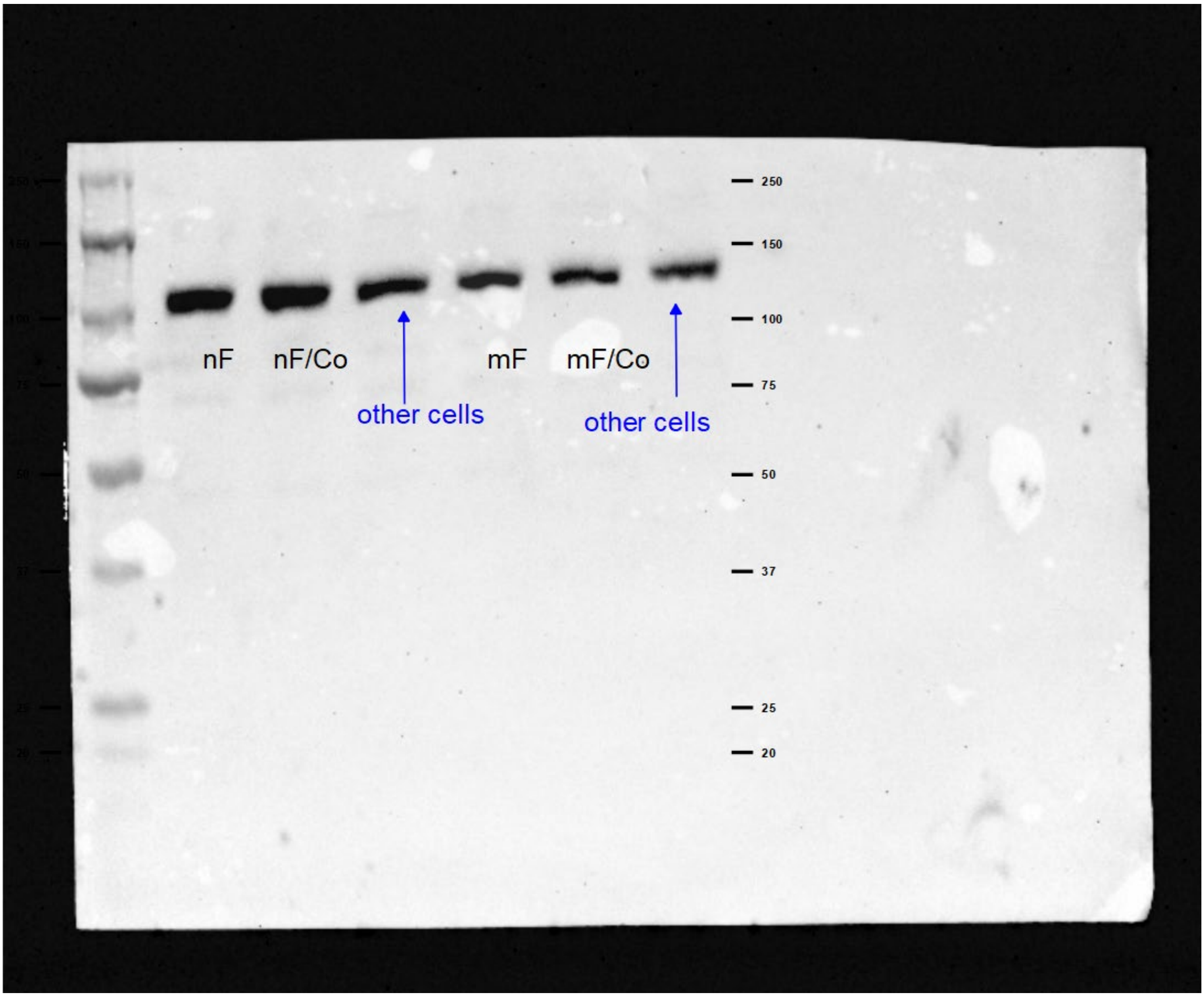

250
150
100
75
50
37
25
20
nF
nF/Co
other cells
mF
mF/Co
other cells

Blot 3 FAP

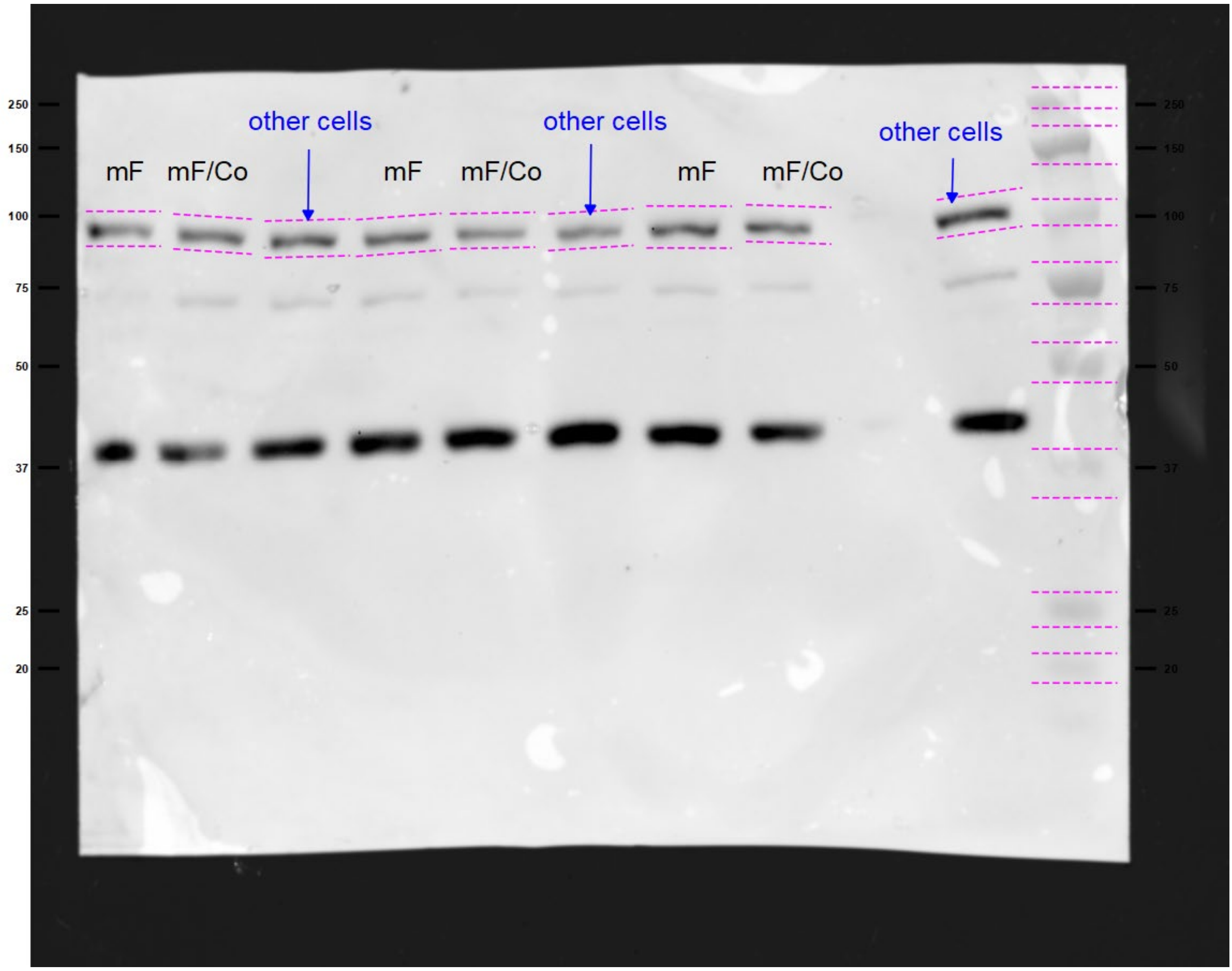
250
150
100
75
50
37
25
20
other cells
other cells
other cells
mF
mF/Co
mF
mF/Co
mF
mF/Co

APC mutation

Blot 1

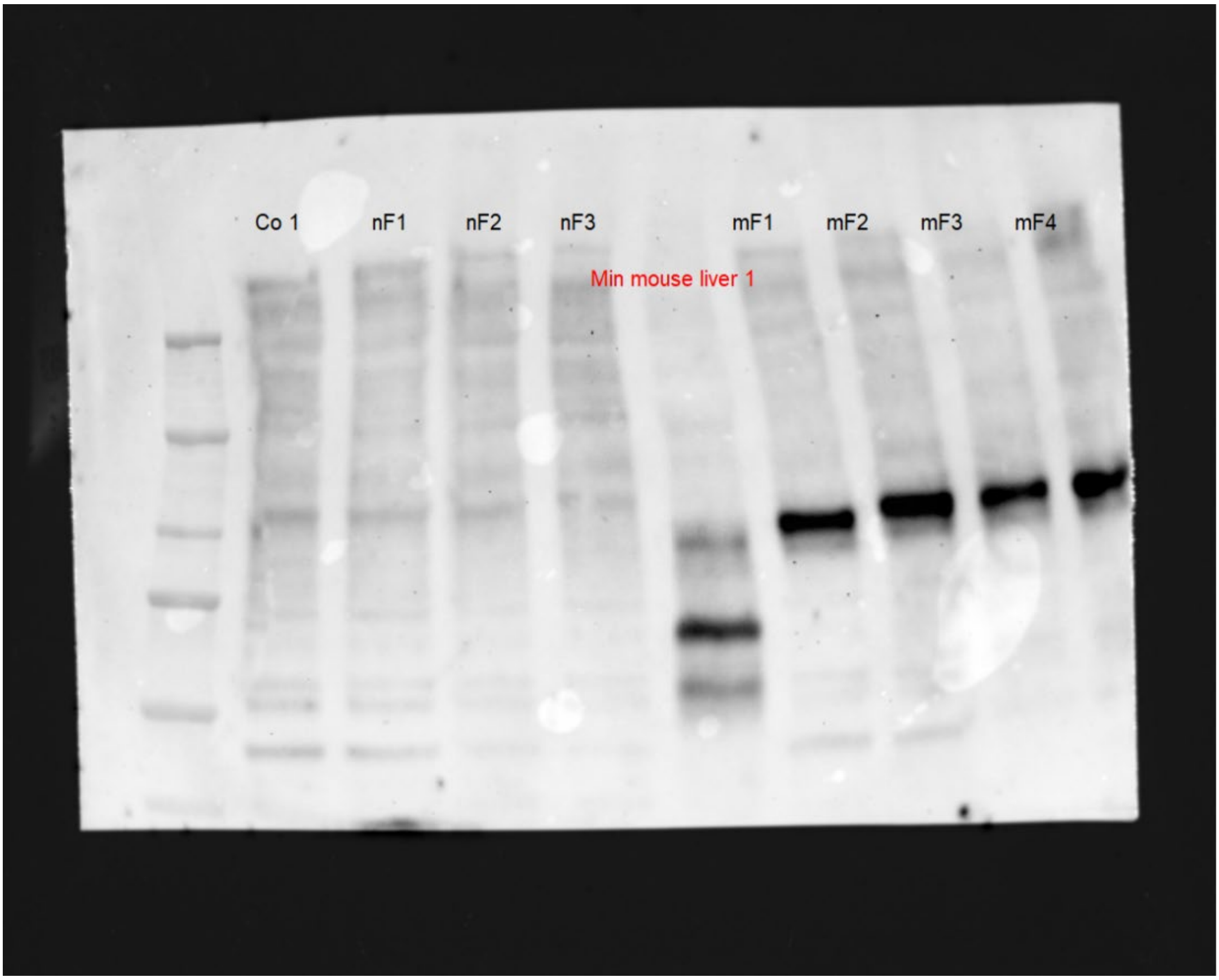

Blot 2

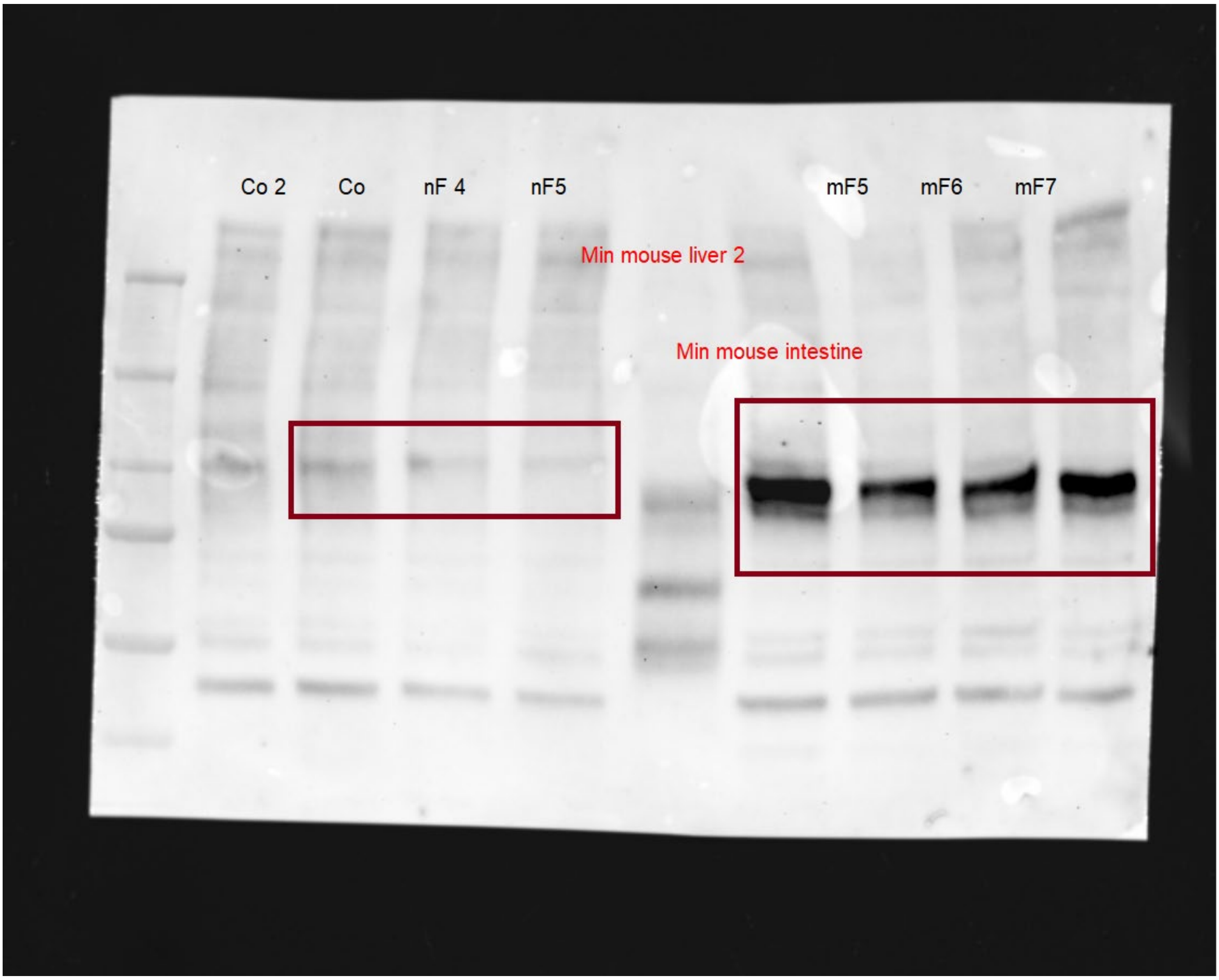
Co 2
Co
nF 4
nF5
mF5
mF6
mF7
Min mouse liver 2
Min mouse intestine

Blot 3

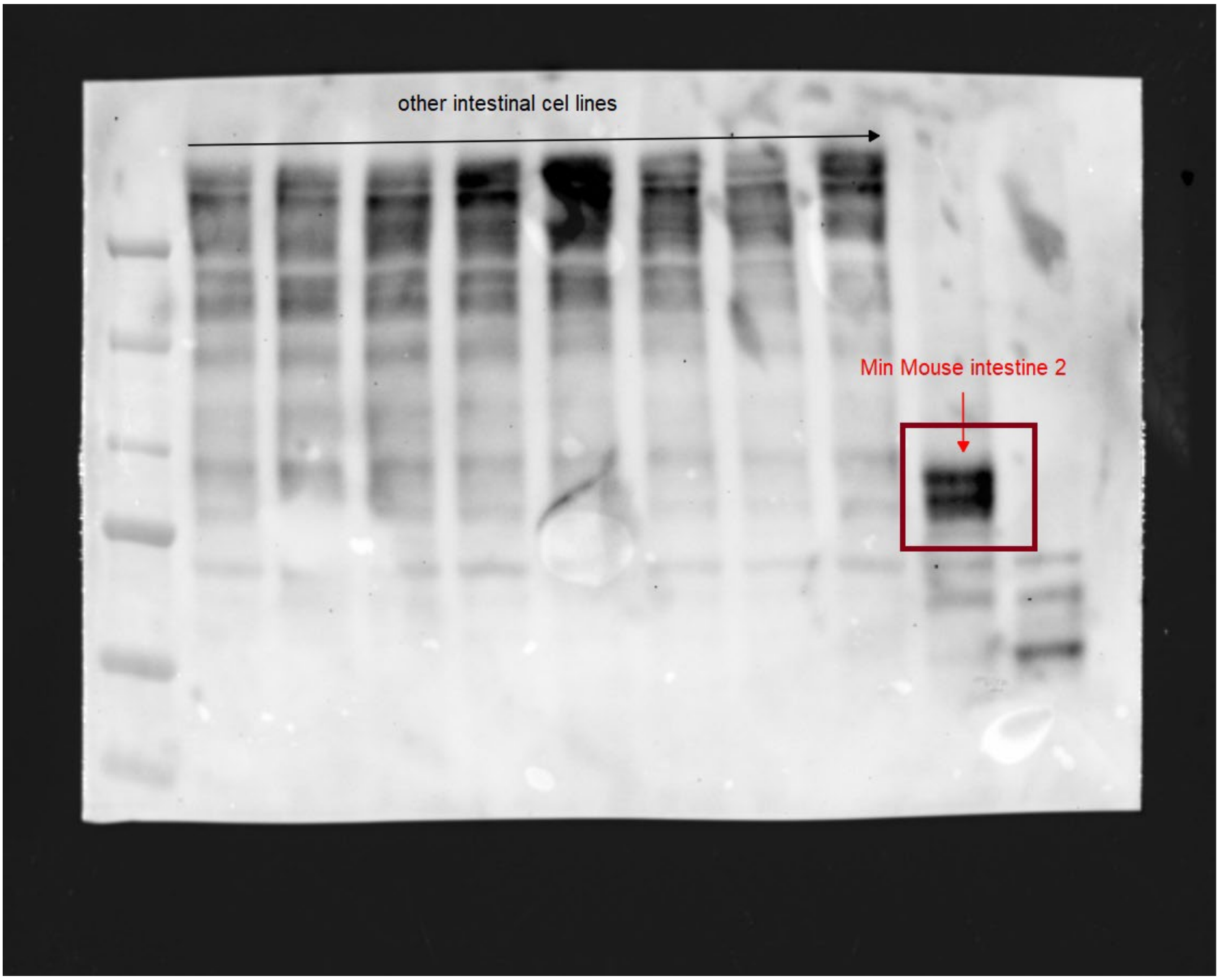

Supplement: S1 Raw images — (PDF) [file pone.0273858.s013.pdf]
